# Ruthenium complexes show potent inhibition of AKR1C1, AKR1C2, and AKR1C3 enzymes and anti-proliferative action against chemoresistant ovarian cancer cell line

**DOI:** 10.3389/fphar.2022.920379

**Published:** 2022-08-11

**Authors:** Jakob Kljun, Renata Pavlič, Eva Hafner, Tanja Lipec, Sara Moreno-Da Silva, Primož Tič, Iztok Turel, Tomaž Büdefeld, Jure Stojan, Tea Lanišnik Rižner

**Affiliations:** ^1^ Department of Chemistry and Biochemistry, Faculty of Chemistry and Chemical Technology, University of Ljubljana, Ljubljana, Slovenia; ^2^ Institute of Biochemistry, Faculty of Medicine, University of Ljubljana, Ljubljana, Slovenia; ^3^ Faculty of Chemical Sciences, Universidad Complutense de Madrid, Madrid, Spain

**Keywords:** ruthenium complexes, synthesis, crystal structure, anticancer, aldo-keto reductase, enzyme inhibition

## Abstract

In this study, we present the synthesis, kinetic studies of inhibitory activity toward aldo-keto reductase 1C (AKR1C) enzymes, and anticancer potential toward chemoresistant ovarian cancer of 10 organoruthenium compounds bearing diketonate (**1**–**6**) and hydroxyquinolinate (**7**–**10**) chelating ligands with the general formula [(η^6^-*p*-cymene)Ru(chel)(X)]^n+^ where chel represents the chelating ligand and X the chlorido or pta ligand. Our studies show that these compounds are potent inhibitors of the AKR enzymes with an uncommon inhibitory mechanism, where two inhibitor molecules bind to the enzyme in a first fast and reversible step and a second slower and irreversible step. The binding potency of each step is dependent on the chemical structure of the monodentate ligands in the metalloinhibitors with the chlorido complexes generally acting as reversible inhibitors and pta complexes as irreversible inhibitors. Our study also shows that compounds **1**–**9** have a moderate yet better anti-proliferative and anti-migration action on the chemoresistant ovarian cancer cell line COV362 compared to carboplatin and similar effects to cisplatin.

## 1 Introduction

Human members of aldo-keto reductase (AKR) subfamily 1C have important roles in many physiological and pathophysiological processes. The enzymes AKR1C1–AKR1C3 are NADPH-dependent reductases with a variety of endogenous and exogenous substrates (Rižner and Penning, 2014). These enzymes play a role in steroid hormone metabolism and act to varying degrees as 3-keto, 17-keto, or 20-keto steroid reductases. In this way, they control the concentrations of active androgens, estrogens, and progestagens which are ligands for the corresponding nuclear receptors. The AKR1C enzymes are thus involved in pre-receptor regulation, with AKR1C1 catalyzing the inactivation of progesterone, AKR1C2 catalyzing the inactivation of 5α-dihydrotestosterone, and AKR1C3 catalyzing the formation of active testosterone and estradiol and are thus implicated in hormone-dependent diseases (Rižner and Penning, 2014). The AKR1C enzymes also play a role in the production of active neurosteroids by catalyzing the 3-keto steroid reduction of 5α-dihydroprogesterone to the most potent allosteric modulator of the γ-aminobutyric acid type receptor allopregnenalone and to the less potent 20α-hydroxy-metabolite (Higaki et al., 2003). AKR1C3 is also known as prostaglandin F2α synthase, as it forms PGF2α and 9α, 11β-PGF2, both of which indirectly activate MAPK kinases and inhibit PPARγ, which can lead to cell proliferation (Penning and Byrns, 2009; Sun et al., 2016). AKR1C enzymes also convert the genotoxic and cytotoxic product of lipid peroxidation, e.g., 4-hydroxy-2-nonenal, into less active metabolites and are thus involved in antioxidant defense (Burczynski et al., 2001), but also in resistance to oxidative stress triggered by chemotherapy.

AKR1C enzymes have been linked to chemo-resistance in different cancers (Penning et al., 2021) as several microarrays and qPCR studies reported upregulation of AKR1C genes in cisplatin/carboplatin- and doxorubicin/daunorubicin-resistant cancer cell lines and tissue samples. Studies in model cell lines also confirmed the association of AKR1C1 and AKR1C2 with resistance to cisplatin, carboplatin, and oxaliplatin in a variety of cancers; ovarian, cervical, lung, colon, oral, head and neck, gastric, and bladder cancers (Deng et al., 2002; Deng et al., 2004; Wang et al., 2007; Chen et al., 2008; Matsunaga et al., 2013; Matsumoto et al., 2016; Zhou et al., 2020; Chen et al., 2013)*,* and AKR1C3 with resistance to anthracycline chemotherapeutics in lung, colon, breast and cervical cancers (Hofman et al., 2014; Zhong et al., 2011). Specific or pan-AKR1C inhibitors have been used in cancer cell lines to restore sensitivity to cisplatin and anthracyclines, with the pan-AKR1C inhibitors flufenamic acid and mefenamic acid also showing efficacy in explant mouse models (Shiiba et al., 2017).

Nowadays drug resistance is the major cause of cancer treatment failure especially in the last stages of the disease. It is a complex phenomenon that includes many underlying mechanisms; changes in the expression of drug efflux pumps and solute uptake transporters, increased drug metabolism, and suppression of cellular stress created by chemotherapeutics. Due to their versatile chemistry, metal compounds offer the possibility to design fine-tuned drugs to overcome this problem (Valente et al., 2021). In our previous studies, we have already tested the potential of different organoruthenium (II) complexes for their efficacy toward platinum-resistant ovarian cancer cells. We have confirmed that the tested compounds remain highly potent toward platinum-resistant cells and act by a mechanism, that is, not common to platinum agents (Kladnik et al., 2021). In another study, activity of selected ruthenium compounds from our library was tested against the multidrug-resistant COLO 205 and COLO 320 colon cancer cell lines, and significant activity was found for some compounds. The resistance of these cell lines is mediated by the overexpression of the ABC-transporter P-glycoprotein, which pumps out xenobiotics from the cytosol (Pivarcsik et al., 2021). One of the aims of this study was thus to get new insights in this important field of metallodrug design focusing on the inhibition of AKR1C enzymes that have roles in chemoresistance.

Potent AKR1C inhibitors with nM Ki values have already been reported and include compounds from different structural groups. To the best of our knowledge, the most potent AKR1C1 inhibitor is 3-bromo-5-phenylsalicylic acid with Ki value of 4 nm and 21-fold selectivity over AKR1C2 (El-Kabbani et al., 2009). The most potent AKR1C2 inhibitor is 5β-cholanic acid-3-one with 21 nM Ki value (Bauman et al., 2005), and estrone lactone EM1404 is currently the most potent AKR1C3 inhibitor with Ki value of 6.9 nm) (Qiu et al., 2007).

**FIGURE 1:**
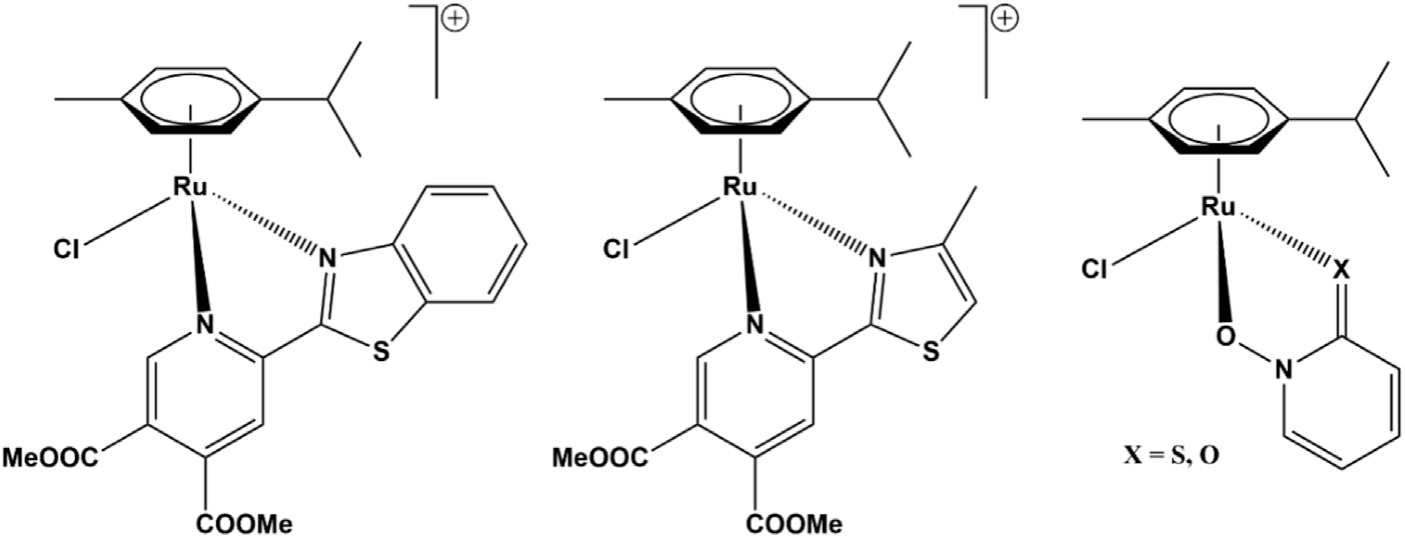
Structures of organoruthenium AKR1C inhibitors. Pan-AKR1C inhibitor (left) and AKR1C3-selective inhibitor (center) (Traven et al., 2015), pyrithione-scaffold-based inhibitors (right) with potent toxicity on hormone-independent breast cancer cell line MCF-7 (Kljun et al., 2016).

We previously reported (Traven et al., 2015; Kljun et al., 2016) that several ruthenium complexes act as specific or pan-AKR1C inhibitors with a combined fast reversible and slow irreversible mechanism of action. An initial study included a series of ruthenium complexes with the general formula [([9]aneS_3_)Ru (dmso-*S*) (NN)]Cl or [(η^6^-*p*-cymene)RuCl(N,N-ligand)]Cl (where [9]aneS_3_ = 1,4,7-trithiacyclononane, dmso-*S* = *S*-bonded dimethylsulfoxide; NN = N,N-donor bipyridine-like ligand) as well as ruthenium precursors and free ligands. One ruthenium complex of the first series (Figure 1, center) and its ruthenium precursor compound [(η^6^-*p*-cymene)Ru (µ-Cl)Cl]_2_ (**P1**) inhibited AKR1C3 with nM Ki values, while its benzothiazole analog inhibited AKR1C1-AKR1C3 with low µM Ki values (Figure 1, left) (Traven et al., 2015). In the next study, we tested four ruthenium complexes of zinc ionophore pyrithione and its oxygen analog 2-hydroxy-pyridine-*N*-oxide and found that organoruthenium complexes (Figure 1, right) inhibited AKR1C1 with sub-µM Ki values, with 2–8-fold higher Ki values for AKR1C3 and 16–40-fold higher Ki values for AKR1C2 (Kljun et al., 2016). The pyrithione complex also irreversibly inhibited AKR1C1, with a 3-fold and 25-fold less potent inhibition of AKR1C2 and AKR1C3 observed, respectively. In addition, both the complex and pyrithione itself showed a cytotoxic effect on breast cancer cell line MCF-7 with low µM IC_50_ values.

**FIGURE 2:**
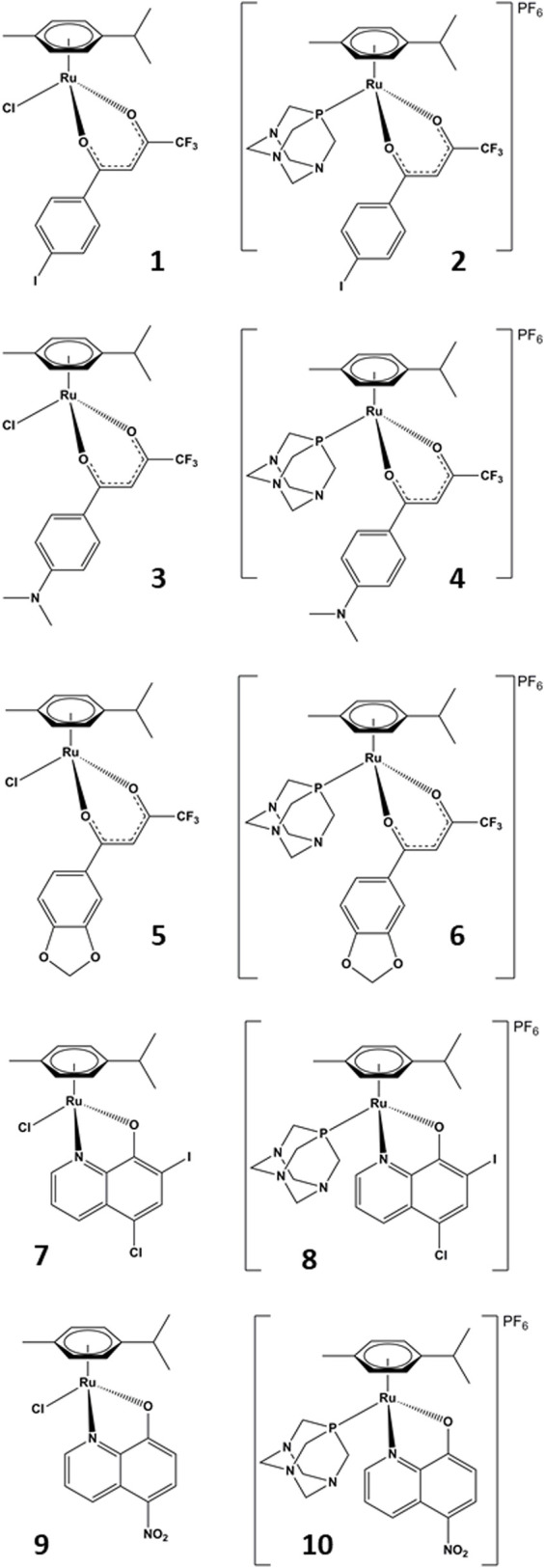
Chemical structures of the studied compounds.

In this study, we investigated the inhibitory action of a series of 10 organoruthenium complexes with β-diketonate and 8-hydroxyquinolinate ligands. Additionally, we also examined their effects on the proliferation and migration of a chemoresistant ovarian cancer cell line. Seven of the studied complexes were chosen from the Turel group compound library and three complexes were newly synthesized to complement the chemical features needed to identify the key structural parameters for the development of novel potent organoruthenium AKR1C inhibitors. The diketonate (compounds **1–6**) and hydroxyquinolinate (compounds **7–10**) scaffolds were chosen to further study the influence of the ruthenium coordination sphere on the AKR1C inhibitory potency and anticancer potential of organoruthenium compounds. In the case of all five chelators, we prepared a pair of complexes where the ruthenium species is allowed direct coordination to the molecular target by including a reactive, fast-releasing chlorido ligand (odd-numbered compounds; Figure 2, left) or is intended to be chemically stable by blocking the last remaining coordination site by phosphine ligand pta (even-numbered compounds; Figure 2, right).

## 2 Materials and methods

### 2.1 Syntheses, characterization, and biological evaluation

[(η^6^-*p*-cymene)RuCl(μ-Cl)]_2_ (**P1**) was purchased from Strem Chemicals, nitroxoline, and the solvents from Sigma-Aldrich. All the materials were used as received. We have previously reported the synthesis of compounds **1** and **2** (Uršič et al., 2017), **5** and **6** (Seršen et al., 2015), **7** (Gobec et al., 2014), **8** (Pivarcsik et al., 2021), and **9** (Mitrović et al., 2019). ^1^H NMR spectra were recorded on a Bruker Avance III 500 spectrometer at room temperature and 500.10 MHz by using TMS as an internal standard. Infrared spectra were recorded with a Perkin–Elmer Spectrum 100 FTIR spectrometer, equipped with a Specac Golden Gate Diamond ATR as a solid sample support. UV–vis spectra were collected on Perkin–Elmer LAMBDA 750 UV/vis/near-IR spectrophotometer. Elemental analyses were recorded using a Perkin–Elmer 2400 II instrument (CHN), and HRMS were measured on an Agilent 6,224 Accurate Mass TOF LC/MS instrument. X-ray diffraction data were collected on an Oxford Diffraction SuperNova diffractometer with Mo/Cu microfocus X-ray source (Kα radiation, λ_Mo_ = 0.71073 Å, λ_Cu_ = 1.54184 Å) with mirror optics and an Atlas detector at 150 (2) K. The structures were solved in Olex^2^ graphical user interface (Dolomanov et al., 2009) by direct methods implemented in SHELXT and refined by a full-matrix least-squares procedure based on F^2^ using SHELXL (Sheldrick, 2015). All non-hydrogen atoms were refined anisotropically. The hydrogen atoms were placed at calculated positions and treated using appropriate riding models. The crystal structures have been submitted to the CCDC and have been allocated the deposition numbers 2063232-2063234.

#### 2.1.1 Synthesis of (η^6^-*p*-cymene)chlorido(4,4,4-trifluoro-1-(4-(dimethylamino)phenyl)-1,3-butandionato)ruthenium(II) (3)

The ruthenium precursor **P1**, the ligand 4,4,4-trifluoro-1-(4-(dimethylamino)phenyl)-1,3-butane, and sodium methoxide (molar ratio 1:2.4:2.2) were suspended in a 10:1 mixture of DCM and methanol and were refluxed 4 h. The solvent was removed using a rotary evaporator and the oily residue redissolved in 5 ml of DCM. The reaction mixture was filtered over a 1 cm layer of silica to remove the precipitated NaCl and unreacted reagents. The silica was further washed with 5 ml of 7:93 MeOH/DCM mixture to completely elute the obtained product. The solvents were removed to obtain an oily residue to which 10–15 ml of dry hexane was added and the orange precipitate of 3 was observed after 20–30 min. The orange product was filtered and dried overnight at 45°C. Amounts: 108 mg RuCYM (0.176 mmol), 110 mg ligand **12** ((2.4 mol eq.; 0.422 mmol) in 21 mg NaOMe (0.387 mmol). Yield: m = 143 mg, η = 76%. Crystals suitable for X-ray diffraction analysis were obtained from an acetone-*d*_*6*_/glycerol 2:1 mixture left standing in an open vial overnight at room temperature.

^**1**^ **H NMR** (500 MHz, CDCl_3_): δ 7.82 (d, *J* = 9.1 Hz, 2H, Ar−H/Ar−H), 6.61 (d, *J* = 9.1 Hz, 2H, Ar−H/Ar−H), 6.14 (s, 1H, H^α^), 5,60 (d, *J* = 5.8 Hz, 1H, Ar−H cym), 5,54 (d, *J* = 5.7 Hz, 1H, Ar−H cym), 5.30 (t, *J* = 6.1 Hz, 2H, Ar−H cym), 3.05 (s, 6H, N(CH_3_)_2_), 2.93 (sept, 1H, Ar−C*H*(CH_3_)_2_ cym), 2.29 (s, 3H, Ar−CH_3_ cym), 1.38 (dd, *J* = 6.9; 4.9 Hz, 6H, Ar−CH(C*H*_3_)_2_ cym).

**IR (cm** ^**−1**^ **, ATR):** 3,041, 2,961, 1,585, 1,550, 1,372, 1,323, 1,293, 1,261, 1,194, 1,141, 1,068, 938, 871, 834, 783, 695.

**ESI-HRMS (CH**_**3**_ **CN)** m/z: [M−Cl]^+^ Exp. 494.0884 Calc. 494.0881.

**Elemental analysis CHN** (%) for C_22_H_25_ClF_3_NO_2_Ru: Calc. C 49.95; H 4.76; N 2.65; Found. C 50.09; H 4.69; N 2.66.

**UV−VIS** (λ [nm] (ε [L mol^−1^ cm^−1^]) c = 0.5 mol/L × 10^–4^ mol/L, MeOH): 258 (8,906), 318 (3,854), 419 (31,470).

#### 2.1.2 Synthesis of (η^6^-*p*-cymene)(4,4,4-trifluoro-1-(4-(dimethylamino)phenyl)-1,3-butandionato)(pta)ruthenium(II) hexafluorophosphate (4)

Complex **3**, ligand pta, and AgPF_6_ (molar ratio 1:1.1:1.1) were suspended in 30 ml of dry acetone in a 50 ml dry round bottom flask and left stirring at room temperature for 48 h. The solvent was removed using a rotary evaporator and the oily residue redissolved in 5 ml of DCM. The reaction mixture was filtered over Celite to remove the precipitated AgCl and unreacted reagents. The reaction mixture was dried with anhydrous sodium sulfate, concentrated to a few mL and 10 ml of dry heptane was used to precipitate **4** as a yellow powder. The yellow product was filtered and dried overnight at 45°C. Amounts: Complex **3**–50 mg (0.094 mmol), 16.4 mg pta (0.104 mmol), 26.4 mg AgPF_6_ (0.104 mmol). Yield: m = 65 mg, η = 86%. Crystals suitable for X-ray diffraction analysis were obtained by layering an acetone solution in a 10 ml test tube with heptane and leaving it to slowly evaporate over 3 days.

^**1**^ **H NMR** (500 MHz, Acetone-*d*_6_) δ 7.95 (d, *J* = 9.2 Hz, 2H, Ar−H/Ar−H), 6.81 (d, *J* = 9.2 Hz, 2H, Ar−H/Ar−H), 6.55 (s, 1H, H^α^), 6.30 (d, *J* = 6.0 Hz, 1H, Ar–H cym), 6.21 (d, *J* = 6.1 Hz, 1H, Ar–H cym), 6.18 (t, *J* = 6.3 Hz, 2H, Ar–H cym), 4.60–4.52 (m, 6H, −NCH_2_N−pta), 4.40–4.31 (m, 6H, −PCH_2_N−pta), 3.14 (s, 6H, −N(CH_3_)_2_), 2.72 (sept, *J* = 13.9, 7.0 Hz, 1H, Ar–C*H*(CH_3_)_2_ cym), 2.09 (s, 3H, Ar−CH_3_ cym), 1.33 (dd, *J* = 20.1; 6.9 Hz, 6H, Ar−CH(C*H*_3_)_2_ cym).

**Selected IR peaks (cm** ^**−1**^ **, ATR):** 2,927, 1,571, 1,542, 1,321, 1,292, 1,261, 1,191, 1,150, 1,131, 1,011, 976, 946, 939, 835, 790, 739.

**ESI-HRMS (CH**_**3**_ **CN)** m/z: [M– PF_6_]^+^ Exp. 651.1654; Calc. M^+^ 651.1650; [M−pta– PF_6_]^+^ Exp. 494.0883; Calc. 494.0881.

**Elemental analysis CHN** (%) for C_28_H_37_F_9_N_4_O_2_P_2_Ru: Calc. C 42.27; H 4.69; N 7.04; Found C 41.82; H 4.52; N 6.86.

**UV**–**VIS** (λ [nm] (ε [L mol^−1^ cm^−1^]) c = 0.5 × 10^–4^ mol/L, MeOH): 314 (3,536), 429 (20,410).

#### 2.1.3 Synthesis of (*η* ^6^-*p*-cymene)(nitroxolinato)(pta)ruthenium(II) hexafluorophosphate (10)

(*η* ^6^-*p*-Cymene) (nitroxolinato)chloridoruthenium (II) (**9**) and silver hexafluorophosphate in a molar ratio of 1:1.2 were suspended in methanol in a brown round-bottom flask and refluxed for 20 min. A 1.5 M equivalent amount of pta was first dissolved in 10 ml of CHCl_3_ and slowly added to the reaction mixture and the reaction mixture was further refluxed for 2 h. The solvents were removed using a rotary evaporator, the oily residue redissolved in DCM and the reaction mixture was filtered over Celite to remove the precipitated AgCl. The clear solution was concentrated to 5–10 and 10 ml of cold *n*-hexane was added to precipitate **10**. The yellow products were filtered and dried overnight at 45°C. Amounts: Complex **9**–30 mg (0.065 mmol), 17 mg pta (0.098 mmol), 20 mg AgPF_6_ (0.078 mmol). Yield: m = 34.6 mg, η = 73%

^**1**^ **H NMR** (500 MHz, Aceton-*d*) δ 9.49 (d, *J* = 9.0 Hz, 1H, C^2^ *H*), 9.08 (d, *J* = 5.0 Hz, 1H, C^4^ *H*), 8.59 (d, *J* = 9.2 Hz, 1H, C^6^ *H*), 7.91 (dd, *J* = 9.0, 5.0 Hz, 1H, C^3^ *H*), 6.99 (d, *J* = 9.2 Hz, 1H, C^7^ *H*), 6.37–6.29 (m, 3H, Ar-*H* cym), 6.15 (d, *J* = 6.1 Hz, 1H, Ar-*H* cym), 4.47–4.36 (m, 6H, pta), 4.15–3.99 (m, 6H, pta), 2.91 (sept, *J* = 14.0, 6.9 Hz, 1H, Ar-C*H*(CH_3_)_2_ cym), 2.40 (s, 3H, Ar-C*H*_3_ cym).

**Selected IR peaks** (cm^−1^, ATR): 3,633, 3,068, 3,054, 2,964, 2,930, 2,658, 1,598, 1,564, 1,506, 1,479, 1,461, 1,413, 1,383, 1,276, 1,244, 1,186, 1,142, 1,096, 1,049, 1,012, 971, 946, 894, 844, 816, 802, 788, 741, 706, 674, 652, 613.

**ESI-HRMS** (CH_3_CN) m/z [M—pta—PF_6_]^+^, Exp 425,0439. Calc. 425,0441 [M—PF_6_]^+^ Exp. 582,1208. Calc. 582,1211.

**UV/Vis** (λ [nm] (ε [L mol^−1^ cm^−1^]) c = 1 mol L^−1^ × 10^−4^ mol L^−1^, CHCl_3_: 274 (21,400), 360 (11,500), 457 (22,100).

**Elemental analysis CHN** (%) for C_25_H_31_F_6_N_5_O_3_P_2_Ru: Calc. C 41.33; H 4.30; N 9.64; Found C 40.99; H 4.62; N 9.61.

### 2.2 Crystal structures of compounds 3, 4, and 10

**FIGURE 3:**
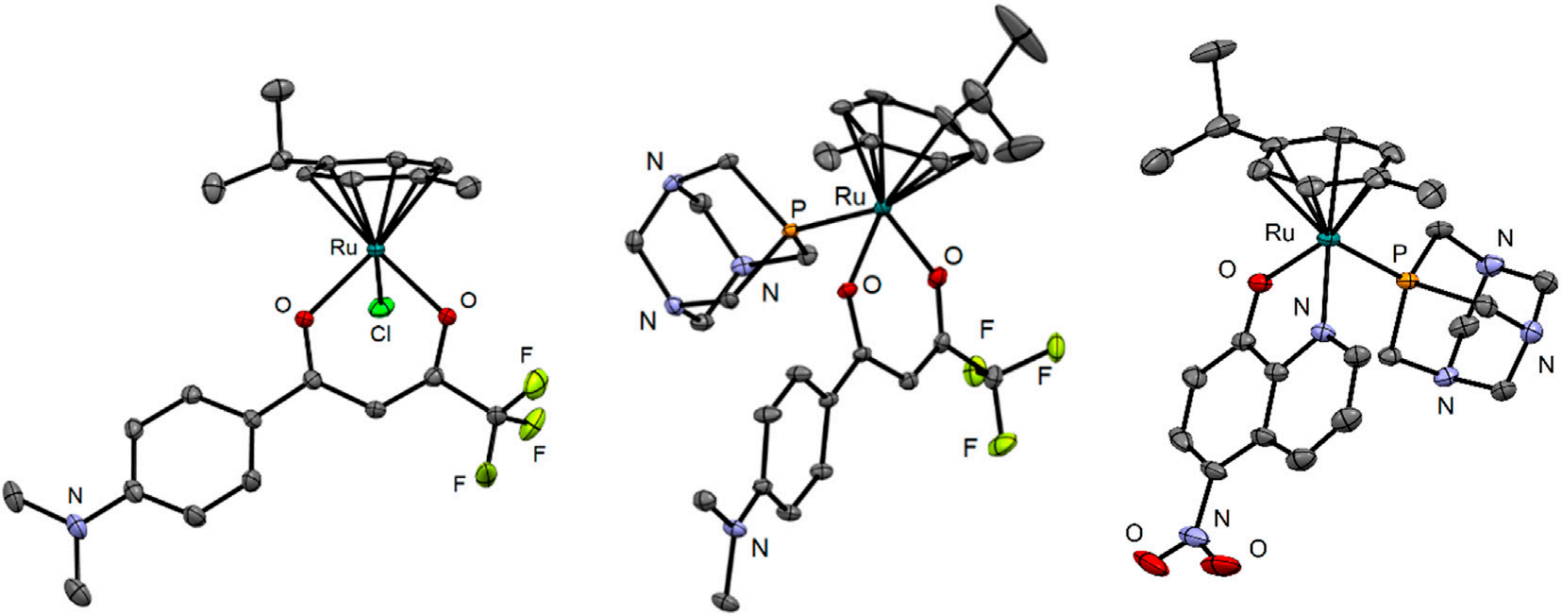
Crystal structures of compounds **3**, **4**, and **10**. The ellipsoids are drawn at 35, 25, and 35% probability levels, respectively. The hydrogen atoms and the PF_6_^–^ counterion in the structures of **4** and **10** as well as the diethyl ether solvate in **10** are omitted.

The crystal structures show the expected piano-stool conformation in all three analyzed organoruthenium complexes (Figure 3). While complex **3** is neutral, complexes **4** and **10** were synthesized and crystallized as hexafluorophosphate salts. The crystal structure of **10** also shows the presence of a co-crystallized diethyl ether molecule which was used as an antisolvent in the crystallization experiment. Crystallographic data and photographs of the analyzed crystals are given in the SI (Supplementary Table S1; Supplementary Figure S1).

### 2.3 Inhibition assays for the AKR1C1–3 enzymes

**FIGURE 4:**
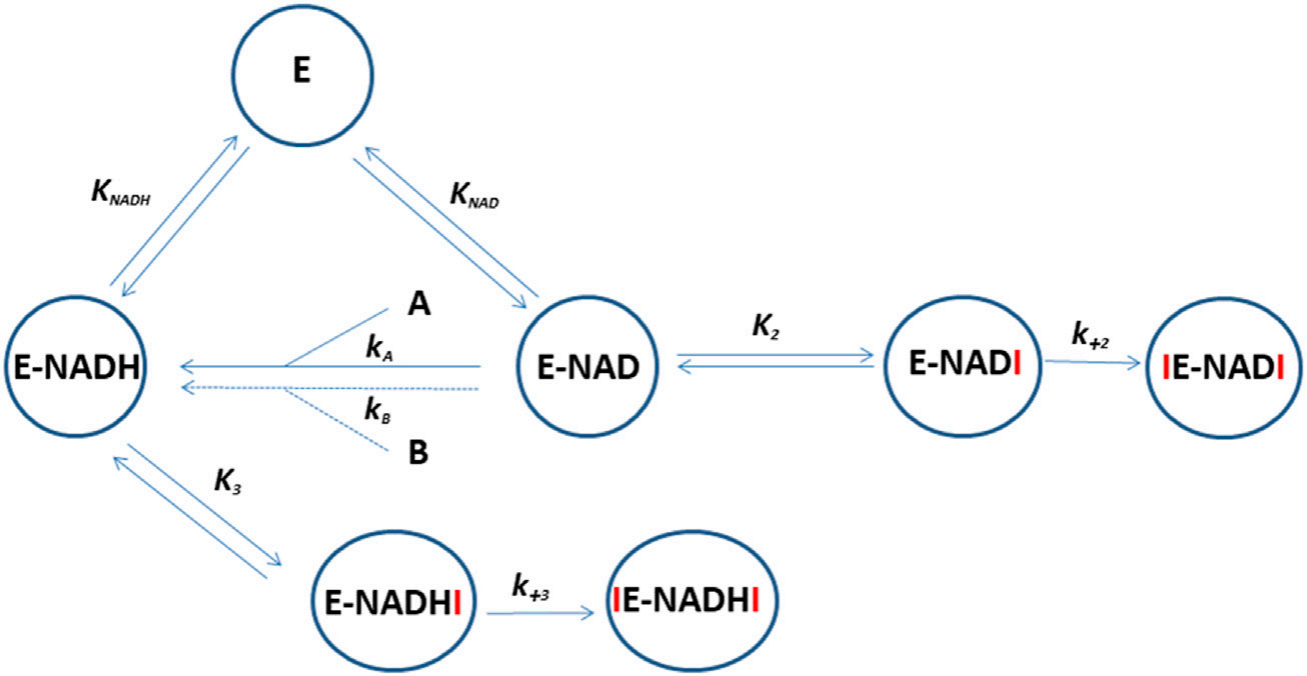
Reaction scheme for the oxidation of 1-acenaphtenol by AKR1C1-3 and its inhibition by ruthenium compounds. E represents apoenzyme, E-NAD and E-NADH represent holoenzyme with bound NAD^+^ and NADH, respectively. A and B are enantiomers of substrate 1-acenaphtenol. Symbols which include I (ruthenium compound) represent holoenzyme in complex with one or two inhibitor molecules. K_NAD_, K_NADH_, and K_2-3_ are equilibrium constants and k_+2–3_ are second-order association rate constants for the binding of the second inhibitor molecule to the enzyme. The progress curves for each enzyme in the presence of ruthenium complexes were analyzed by the WEB server ENZO (https://enzo.cmm.ki.si/) designed exactly for such purposes (Bevc et al., 2011).

To investigate the effect of ten selected ruthenium compounds, we measured their effect on the time course of oxidation of the artificial substrate 1-acenaphtenol by recombinant enzymes AKR1C1–3. The recombinant enzymes AKR1C1–AKR1C3 were prepared as described previously (Brozic et al., 2006). Since the artificial substrate is racemic, the oxidation follows a double exponential pattern as described previously (Traven et al., 2015). The *in vitro* catalytic activity of AKR1C1–3 enzymes was determined spectrophotometrically by monitoring the increase in NADH absorbance at 340 nm (ε_(λ340)_ = 6220 M^−1^cm^−1^) in the presence of the substrate 1-acenaphthenol. Enzymatic reactions (300 µL) were performed in 100 mm potassium phosphate buffer (pH 9.0), 0.005% (v/v) TritonX-114, 5% (v/v) DMSO, containing 2.3 mm NAD^+^ and 1-acenaphthenol at final concentrations of 90, 180, and 250 µM for AKR1C1, AKR1C2, and AKR1C3, respectively. A total of 5 µL of a tested compound in DMSO was added to the reaction mixture. The final concentrations of the tested compounds ranged from 1 to 200 μM, depending on the extent of inhibition (Supplementary Data). The reaction was started by adding 15 µL of AKR1C1, AKR1C2, or AKR1C3 dissolved in 1x PBS (pH 7.3) at final concentrations of 0.11, 0.16, and 1.5 µM, respectively. AKR1C1–3 activity was measured at 37°C for 3 h without shaking using a PowerWave XS microplate reader (Biotek; Winooski, VT, United States). Measurements at seven different concentrations were performed in duplicates or triplicates (Supplementary Figures S2). To mechanistically explain all the obtained progress curves for each ruthenium complex on each enzyme by a common reaction scheme, we extended the originally proposed scheme [32] so that the interaction of the ruthenium complex with apo- and holoenzyme was allowed to be rapid and followed by a slow irreversible association of a second ruthenium complex molecule. However, the affinity of the Ru complexes for the apoenzymes was much lower so this part was excluded from the proposed kinetic model for clarity (Figure 4).

### 2.4 Visualization of the enzyme-inhibitor complex

**FIGURE 5:**
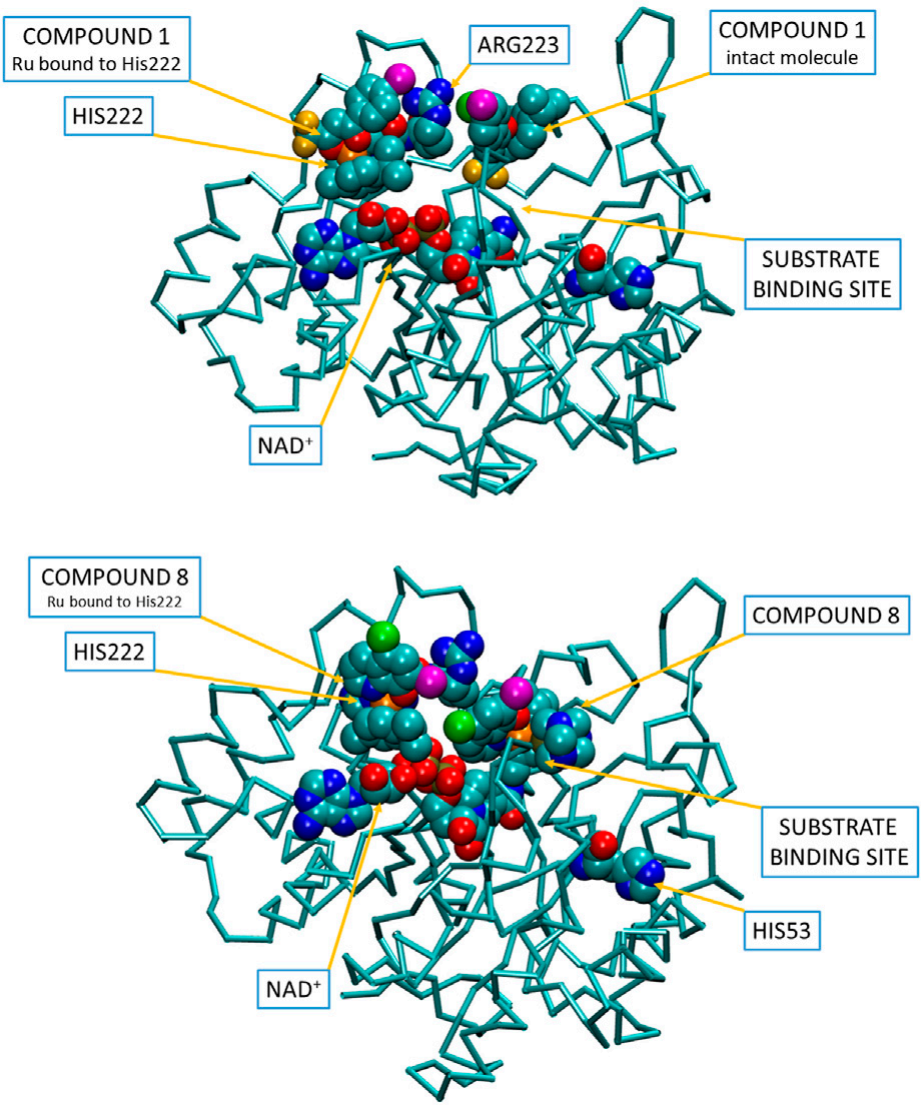
Visualization of a putative binding of two molecules of compounds **1** and **8** into the open conformation of holo-AKR1C1, corroborating the proposed reaction scheme shown in Figure 4. Inhibitor and substrate molecules are depicted in spacefill (carbon—cyan, oxygen—red, nitrogen—blue, fluorine—yellow, iodine—purple, chlorine—green, ruthenium—orange, phosphorus—pale yellow). Video visualizations of molecular dynamics simulations of binding of compounds **1** and **8** can be found as Supplementary Data.

To visualize the interactions for the binding of two Ru complexes (**1** and **8**; Figure 5) to AKR1C enzymes in accordance with the general reaction scheme, we first submitted 3D models of the three substituents to the ParamChem, a web server for the automatic generation of additive force fields in CHARMM (CGenFF). Subsequently, the AKR1C1 coordinates (PDB code 3NTY, devoid of all hetero atoms) with docked one intact complex **1** or **8** molecule as well as the fragment without the respective monodentate ligand (proposed active species) were submitted to Charmm-gui server invoking solution builder module. In the submitted structure, the intact complex **8** molecule was put on the substrate-binding position in the active site while the second one, devoid of pta ligand, was put with its ruthenium ion within 2.5 A distance from Nε2 atom of His222. Similarly, chloride was substituted by His222 in the case of complex **1**. In the Charmm-gui input script, we then combined the obtained stream files with the latest version of the all-atom CGenFF additive parameter set and manually adopted VDW and the electrostatic parameters for the Ru(II) cation, using the crystal structure as a topology standard. During the equilibration and all production dynamic simulation runs (100 ns) we restrained the ruthenium ion with its ligands from the complex **1** or **8** crystal structure at their particular distances. No restraint was applied to the bond between Ru(II) and the Nε2 His222.

### 2.5 Cell lines

The cell line COV362 (CVCL_2,420) was originally established from a high-grade ovarian serous adenocarcinoma from a metastatic site in the pleural effusion (van den Berg-Bakker et al., 1993) and was purchased from ECACC (ECACC 07071910) as p37 on 13 October 2017. The growth medium for COV362 cells was DMEM (D5546; Sigma–Aldrich GmbH), with 10% FBS (F9665; Sigma–Aldrich GmbH) and 2 mM L-glutamine (G7513; Sigma–Aldrich GmbH). COV362 cells in passages p37 + 12 to p37 + 17 were used in this study and seeded at a concentration of 3.5 cells/ml × 10^4^ cells/ml for proliferation studies and at a concentration of 5.3 × 10^5^/ml for migration studies. The cell line OVCAR-4 (CVCL_1,627) is a high-grade serous ovarian adenocarcinoma cell line established from a patient refractory to cisplatin (Johnson et al., 1997). The cell line was purchased from Merck on 19 March 2021. The growth medium for OVCAR-4 cells was RPMI-1640 (Sigma R5586) supplemented with 2 mM glutamine (G7513; Sigma–Aldrich GmbH), 10% FBS (F9665; Sigma–Aldrich GmbH), 0.25 μg/ml insulin (I9278; Sigma–Aldrich GmbH). OVCAR-4 cells in passages p+6 to p+15 were used in this study and seeded at a concentration of 6 cells/ml × 10^4^ cells/ml. The control cell line HIO80 (CVCL_E274) was obtained from Andrew K. Godwin (University of Kansas Medical Center, Kansas, United States) on 20 October 2017 as p+72. It was originally established from ovarian surface epithelium (Yang et al., 2004). HIO80 cells were grown in RPMI medium (R5886, Sigma–Aldrich GmbH) supplemented with 10% FBS (F9665; Sigma–Aldrich GmbH), 2 mm L-glutamine (G7513; Sigma–Aldrich GmbH) and 6.6 µg/ml insulin (I9278; Sigma–Aldrich GmbH). HIO80 cells in passages p72 + 8 to p72 + 19 were used in this study and seeded at a concentration of 2 × 10^4^ cells/mL. HIO80 in passage +10 was authenticated by short tandem repeats (STR) profiling performed by ATCC on 22 February 2019. Cells were grown in a humidified atmosphere containing 5% CO_2_ at 37°C. All cell lines were negative for mycoplasma infection which was periodically tested using MycoAlert^TM^ mycoplasma detection kit (Lonza, Basel, Switzerland).

### 2.6 Cell proliferation and invasion assays

The effect of ruthenium compounds on the proliferation of ovarian cancer cell lines COV362 and OVCAR-4 and the control cell line HIO80 was analyzed using the cell proliferation reagent alamarBlue HS (A50101; Thermo Fisher Scientific) following the manufacturer’s instructions.

Cells were seeded in 96-well plates in 100 µL of medium and allowed to adhere and grow for 24 h. Compounds **1–10** were dissolved in DMSO except for compound **7**, which was dissolved in DMF due to poor solubility in DMSO. Cisplatin and carboplatin purchased from Sigma–Aldrich GmbH (cat. number: PHR1624, lot: LRAB7778) and (cat. number: C2538, lot: MKCK1243) were dissolved in sterile water. Working solutions were prepared by a serial dilution of stock solutions with a culture medium. After the initial incubation, cells were treated with ruthenium complexes, cisplatin, and carboplatin. A range of final concentrations between 0.001 and 300 µM were used for ruthenium complexes and cisplatin, and between 0.001 µM and 1 mM for carboplatin. After 48 h of incubation, plates were incubated with 20 µL of alamarBlue. After 4 h absorbance was measured with a BioTek microplate spectrophotometer at 570 nm, with the reference wavelength set at 600 nm. First, the background value of a well containing the active substance without cells was subtracted, then the average values for duplicates/triplicates were calculated. The absorbance of the well containing only untreated cells was used as a normalization control. The half-maximal effective concentration (IC_50_) was determined for all ruthenium complexes with a growth-inhibiting effect on cells by constructing a dose-response curve (Graph Pad Prism, Version 8.0).

Cell migration assay was performed using Ibidi^®^ three-well culture inserts. The inserts were placed in six-well plates. In total, 70 µL of cell suspension was seeded in the inserts and allowed to adhere and grow for 24 h. When the cells formed a confluent monolayer, the inserts were carefully removed and the cells were washed two times with DPBS. Cells were treated with 100 μM (final concentration) of compounds **8**, **9**, cisplatin, and carboplatin in 2 ml of growth medium. Cells treated with a medium containing the same volume of sterile water or DMSO were used as the controls. Images of cell gaps were taken with Axio Scope A1 Polarized Light Microscope (Zeiss) at the time of treatment, 24 and 48 h after, and analyzed using ImageJ software.

## 3 Results and discussion

### 3.1 General considerations and complex syntheses

Ruthenium compounds bearing ligands of these two large and chemically diverse ligand families have previously been investigated by our group (Uršič et al., 2017; Mitrović et al., 2019; Kljun and Turel, 2017). We found that organoruthenium compounds in general act as multimodal agents. Their anticancer properties are the result of a combination of factors. They can cause systemic cellular damage by the generation of ROS species which induces apoptosis (Seršen et al., 2015; Kljun et al., 2018; Ruiz et al., 2019), and they disrupt redox homeostasis by acting on sulfur-containing molecules in the glutathione cycle (Briš et al., 2019) and they can act on specific enzymes involved in molecular mechanisms of cancer development, progression, and resistance such as aldo-keto reductases (Traven et al., 2015) (Kljun et al., 2016) or formation of metastases such as cathepsin B (Mitrović et al., 2019). The nature of the interaction with protein targets can be modulated by the presence or absence of fast-reacting leaving ligands which was confirmed in several studies involving structurally very diverse molecular targets such as zinc fingers (Sheng et al., 2019) or carbonic anhydrase (Seršen et al., 2019). The complexes were synthesized according to procedures published in the above-cited papers. Generally, the dimeric ruthenium precursor was reacted with 0.5 M equivalents of the appropriate ligand in presence of sodium methoxide as a base to yield the chlorido complexes. The reactions are robust and proceed in good yields in chlorinated solvents (chloroform or dichloromethane) or acetone. A slight excess of the ligand is used if TLC reveals the presence of the unreacted ruthenium precursor (R_f_ = 0, red color). A small amount of methanol can be used if the ligands are not sufficiently soluble. The reaction mixture is then concentrated and redissolved in 2–3 ml of chosen solvent. Side products and unreacted reagents are removed by filtration through a thin layer of silica. The products are precipitated by the addition of cold *n*-hexane or heptane. Pta complexes are then prepared by reacting chlorido compounds with a slight excess of silver salt AgPF_6_ and pta using chlorinated solvents or acetone as the reaction medium. These reactions are more susceptible to side reactions and some care must be taken to ensure that solvents and glassware are dried before use and both the silver salts and pta are stored in dry conditions. Reactions are slower and take 24–72 h at room temperature; however, the progress can easily be monitored by TLC. Heating the reaction mixtures results in the appearance of multiple side products. The compounds also decompose on silica and Celite must be used for the removal of AgCl. Precipitation often occurs only overnight in refrigerated solutions.

### 3.2 Ruthenium complexes act as inhibitors of AKR1C1–AKR1C3 enzymes

In this study, five pairs of ruthenium complexes were examined as inhibitors of recombinant enzymes AKR1C1–AKR1C3. The proposed reaction scheme indicates that Ru complexes can bind to the AKR1C holoenzymes E-NAD^+^ or E-NADH in a reversible manner followed by the binding of an additional molecule in an irreversible manner (Figure 4) as previously proposed for other Ru complexes (Traven et al., 2015; Kljun et al., 2016). However, complex **9** showed virtually no irreversible inhibition of AKR1C1–AKR1C3 and complex **10** showed no irreversible inhibition of AKR1C3. All Ru complexes, with the exception of outlier **9**, which showed a very weak inhibitory effect against AKR1C1–AKR1C3, acted preferentially as inhibitors of AKR1C1 and showed less effect against AKR1C2 and AKR1C3. Almost no inhibition of AKR1C3 was observed for **7** and **10** (Table 1).

**TABLE 1:** Inhibition and rate constants for complexes 1–10.

	AKR1C1				AKR1C2				AKR1C3			
Cpd	K_2_ (µM)	k_+2_ (M^−1^ s^−1^)	K_3_ (nM)	k_+3_ (M^−1^ s^−1^)	K_2_ (µM)	k_+2_ (M^−1^ s^−1^)	K_3_ (nM)	k_+3_ (M^−1^ s^−1^)	K_2_ (µM)	k_+2_ (M^−1^ s^−1^)	K_3_ (nM)	k_+3_ (M^−1^ s^−1^)
**1**			21.4	9.4	305		52.7	7.7	105		163	0.9
**2**	2.8	25.9			80	12.6			116	5.3		
**3**	107	31.5	42.9	4.3	157	7.4	1,125	55.4	179	10.2	306	1.2
**4**	86.9	1.4			340	1.9			441	1.8		
**5**			23.5	6.0			66.2	7.3			150	0.5
**6**	3.1	48.8			8.3	172			92.7	90		
**7**			345	17.1			65.4	11.1			*	
**8**	0.35	2,943			1.6	929			**			
**9**	*227*				*191*				*439*			
**10**	49.7	13.5			42.5	5.1			*561*			

*Compound **7** shows parabolic inhibition with one irreversible rate constant k_+3_/K_3_ = 1.63.10^6^ M^−2^ s^−1^.

**Compound **8** shows parabolic inhibition with one irreversible rate constant k_+3_/K_3_ = 1.97.10^6^ M^−2^ s^−1^. Compounds **9** and **10** show virtually no irreversible inhibition and their dissociation constants are written in *italics*.

The analyses were performed using two experimental data sets simultaneously. AKR1C1: The dissociation constants for the binding of coenzymes and second-order specificity constants for both substrate enantiomers for the reaction in the absence of ruthenium compound were: K_NAD_ = 0.16 mm, K_NADH_ = 0.61 mm, k_spec1_ = 16,920 M^−1^ s^−1^, k_spec2_ = 1580 M^−1^ s^−1^. Compound **9** shows virtually no irreversible inhibition and its dissociation constant is written in *italics*. AKR1C2: The dissociation constants for the binding of coenzymes and second-order specificity constants for both substrate enantiomers for the reaction in the absence of ruthenium compound were: K_NAD_ = 0.16 mm, K_NADH_ = 0.61 mm, k_spec1_ = 3000 M^−1^ s^−1^, k_spec2_ = 140 M^−1^ s^−1^. Compound 9 shows virtually no irreversible inhibition and its dissociation constants are written in *italics*. AKR1C3: The dissociation constants for the binding of coenzymes and second-order specificity constants for both substrate enantiomers for the reaction in the absence of ruthenium compound were: K_NAD_ = 0.16 mm, K_NADH_ = 0.61 mm, k_spec1_ = 230 M^−1^ s^−1^, k_spec2_ = 38 M^−1^ s^−1^.

The kinetic parameters for all Ru complexes (Table 1) revealed that complexes **1**, **5**, **3**, and **7** act as potent reversible inhibitors of AKR1C1, and less of AKR1C2 and AKR1C3, while complexes **8**, **6**, **3,** and **2** act more efficiently as irreversible inhibitors of AKR1C1 and AKR1C2 and less of AKR1C3. We can compare the potencies of Ru complexes for reversible inhibition, by determining inhibition constants K_2_ and K_3,_ which characterize the binding of Ru complexes to holoenzymes E-NAD and E-NADH, respectively. The K_2_ values were in the micromolar range for most complexes for all three AKR1C enzymes, except for inhibition of AKR1C1 by complex **8**, which showed a submicromolar (0.35 µM) K_2_ value. On the other hand, K_3_ values were much lower. The lowest K_3_ value of 21.4, 31.5, and 48.8 nm were determined for the inhibition of AKR1C1 by **1**, **5**, and **3**, respectively. Compared to AKR1C1 compounds **1**, **5**, and **3** showed 2.5-fold, 2.8-fold, and 26-fold less efficient inhibition of AKR1C2, respectively. The inhibition of AKR1C3 by **1**, **5**, and **3**, was even less efficient by 7.6-fold, 6.3-fold, and 7.1-fold, respectively. The most potent reversible inhibitors of AKR1C1 (**1**, **5**, and **3**) bear the chlorido ligand similarly as previously shown for pyrithione-based Ru complexes that acted as submicromolar inhibitors of AKR1C1 (Kljun et al., 2016).

The irreversible binding of the second inhibitor molecule to E-NAD-I and E-NADH-I and subsequent inhibition are characterized by rate constants k_+2_ and k_+3_, respectively (Table 1; Figure 4). The rate constant k_+2_ values were generally higher than k_+3_ values. The highest k_+2_ values of 2943 M^−1^ s^−1^ and 929 M^−1^ s^−1^ were determined for inhibition of AKR1C1 and AKR1C2 by **8**. This was followed by a k_+2_ of 172 M^−1^ s^−1^ for inhibition of AKR1C2 by compound **6**. Experimental data thus show that compounds with pta ligands act as more potent irreversible inhibitors which seems to be in contrast to our previous study (Kljun et al., 2016).

### 3.3 Molecular mechanism of AKR1C1–3 inhibition

With the results of the kinetic studies in hand, we aimed to further understand the molecular mechanism of inhibition. Modeling the interactions of ruthenium compounds with macromolecules is proving to be quite a difficult task due to the general lack of structural data of protein complexes with ruthenium compounds. A quick search of the PDB database for ruthenium ions (as of 4.2.2022) reveals only 44 structures. An analysis of the hits shows a wide structural diversity of ruthenium compounds used in the published studies. It includes conventional octahedral coordination compounds, which are mostly the results of studies with one of the two ruthenium molecules that have reached phase II clinical trials, NAMI-A, and KP1339. Other hits show ruthenocene compounds, cyclopentadienyl ruthenium complexes, CO-releasing ruthenium-carbonyl complexes, and photoactive polypyridyl ruthenium complexes and ruthenium dyes used in biochemical assays. Only five structures show structurally similar organometallic arene-ruthenium compounds with chlorido, pta, and different bidentate ligands as used in the present study, with very different results depending on the experimental conditions and chemical reactivity of the compounds.

For ruthenium compounds bearing a π-bonded arene ligand and a bidentate functional ligand, the chemical reactivity can be modulated by an appropriate choice of the monodentate ligand which can (de)stabilize the oxidation state of the central ruthenium ion and thus affect the stability of the arene ligand. On the other hand, the halide ligands (Cl, Br, I) with the ruthenium ion stable in the +2 oxidation state generally act as fast-reacting leaving groups with decreasing reaction kinetics, which is useful for the design of covalent inhibitors with high binding affinity for intermediate/soft Lewis base amino acid residues such as His or Cys. In the case of histidine, the complexes interact with the exposed nitrogen (either Nε or Nδ1 atom). Substitution of halide ligands with nitrogen heterocyclic ligands (e.g., imidazoles (Legina et al., 2020)) and phosphines such as pta form much more stable, slow-to-react complexes that act through non-covalent interactions, often resulting in reversible inhibitors of varying degrees of potency. However, the inclusion of strong π-acceptor ligands can also lead to the release of the arene ligand to free three coordination sites on the metal ion for direct interaction with the proteins studied (Sullivan et al., 2018). All in all, the little structural data, that is, available in these systems mirrors remarkably well the known chemistry of the ruthenium species, though it obviously suffers from the chemistry equivalent of survivorship bias, i.e., the PDB database contains only the results of experiments with positive outcomes. The protein structural data however suggest that these compounds could potentially free three metal coordination sites by releasing the cymene ligand leading to very slow but potent and irreversible inhibitors bound to up to three amino acids residues. Indeed, in the case of our diketonate and hydroxyquinolinate complexes, in our past studies, we detected trace amounts of cymene release in NMR stability experiments within a few days at room temperature conditions. This can be explained by trans-effect induced Ru-cymene bond labilization by π-acceptor ligands such as phosphines, however, these reactions are generally very slow and considered well outside the timeframe of enzyme kinetic assays as we previously reported in our studies (Kladnik et al., 2019; Kladnik et al., 2021; Pivarcsik et al., 2021). However, the results of the current study point to this process being of great influence in the case of AKR1C inhibition.

In our experiments, we can observe the binding of a first inhibitor molecule which causes rapid inhibition of the holoenzyme. This is followed by a slow irreversible inactivation of the enzyme with the second inhibitor molecule. This reaction course is very consistent and is seen in nine of ten compounds (as well as in previous studies) and is operative in all three enzymes though to varying degrees of potency. Indeed, the individual constants differ from enzyme to enzyme and the compounds show the most potent reversible inhibition of AKR1C1 although AKR1C1-3 enzymes share a high percentage of amino acid sequence identity, from 87 to 98% (Brožič et al., 2011). There is only a seven amino acid difference between AKR1C2 and AKR1C1 (98% identity), and only one residue within the active site (Leu/Val54) but still compounds **1**, **5,** and **3** inhibit preferentially AKR1C1 with 2.5-fold, 2.8-fold, and 26-fold less efficient inhibition of AKR1C2. The lower percentage of identical amino acids is seen between AKR1C1 and AKR1C3 (88.3% identity) and AKR1C2 and AKR1C3 (87.3%) accordingly the compounds, in general, show less activity against AKR1C3.

To understand the two subsequent inhibitory effects of ruthenium compounds one must bear in mind the basic events during the enzymes’ turnover cycle: all three enzymes exist in open conformation and adopt the closed conformation upon binding of the coenzyme and the substrate. It seems that this open conformation is necessary for the binding of the ruthenium compound to the holoenzyme as suggested by the common kinetic reaction scheme. This open conformation can be further stabilized by a reversible binding of the first ruthenium complex, which in turn enhances the possibility of the coordination of a second one. Closing/opening in AKR1C enzymes is predominantly concerning a very flexible loop comprising residues 220–233, but there are also subtle movements in other parts of the molecule. We have suggested previously the target His53, which is more exposed in the open conformation (Kljun et al., 2016). However, there is another histidine, situated exactly in the closing/opening loop: His222. The latter is only accessible from the water environment in the open conformation, so it is a good candidate for the coordination of a ruthenium inhibitor. Moreover, this hypothesis is strongly supported by the fact, that ruthenium complexes affect AKR1C3 very poorly, which has Gln instead of His at this position. On the other hand, His53 is conserved in all three enzymes and appears accessible in open conformation somehow better than in closed conformation.

We have summarized the kinetics of the interactions between ten ruthenium complexes and three AKR1C enzymes in a comprehensive, generally operating, reaction scheme (Figure 4). In accordance with the available crystallographic information, it suggests that NADH together with the first ruthenium complex molecule prepares the enzyme in an open conformation to coordinate a second ruthenium complex molecule.

In the case of chlorido complexes (odd numbered), we propose the coordination of the hydrolyzed species to the His53 (present in all enzymes) or His222 (Figure 5) (present in only AKR1C1 and AKR1C2). For pta complexes (even numbered), we again propose the dissociation of the monodentate ligand for the binding of the second inhibitor molecule. However, the high binding constant is in contrast with the expected mode of action and could point to a slightly different molecular mechanism. Upon close inspection of both the available structural data in PDB and solution speciation studies of these compounds, we could tentatively propose an alternative binding mechanism of the related cymene-free chemical species. Indeed, the molecular dynamics simulation of the corresponding hydrolyzed triaqua species of complex **8** [Ru(H_2_O)_3_ (pta) (cq)]^+^ shows that the proposed species is stable in the enzyme environment (Supplementary Data) and could thus potentially form stable adducts.

### 3.4 Ruthenium complexes show anti-proliferative effects and decrease migration of chemoresistant ovarian cancer cell lines COV362

Our study revealed that Ru complexes act as combined reversible and irreversible inhibitors of recombinant enzymes AKR1C1–AKR1C3. Knowing that AKR1C enzymes, particularly AKR1C1 and AKR1C2, are associated with chemoresistance, we next evaluated their effects on chemoresistant high-grade serous ovarian cancer (HGSOC) cell line. HGSOC are aggressive tumors characterized by high mortality. Currently, only about 30–40% of patients with HGSOC survive 5 years due to the development of resistance to chemotherapeutic treatments, mainly platinum-based drugs, and paclitaxel (Armstrong et al., 2012).

The ruthenium complexes (with the exception of **4**, for which we had problems obtaining samples with sufficient purity during resynthesis) were therefore tested for their effect on the proliferation and viability of a chemoresistant ovarian cancer cell line COV362 and a control ovarian cell line HIO80. All nine complexes tested decreased the proliferation of both cell lines (Table 2). The highest antiproliferative effect against COV362 was observed for compound **7**, with an IC_50_ value of 17.67µM, followed by compounds **9** and **8** with IC_50_ values of 21.53 and 38.04 µM, respectively. Complexes **1** and **3** also showed I C_50_ values below 100 µM. Complexes **2** and **5** were less effective (IC_50_ > 100 µM) and the lowest effect was observed for complexes **6** and **10** with IC_50_ values near and above 200 µM. As expected, the ruthenium complexes showed better efficacy toward the control cell line HIO80, with IC_50_ values < 50 µM obtained for five compounds (Table 2). The effects of the most potent complexes **7**, **8,** and **9** were examined also on less chemoresistant/chemosensitive cell line OVCAR-4. Compound **9** was more efficient on OVCAR-4 compared to COV362 with a 1.5-fold lower IC_50_ value, while compounds **7** and **8** showed weaker anti-proliferative action against OVCAR-4 as compared to COV362 with 1.6 and 2.6-fold higher IC50 values, respectively.

**TABLE 2:** IC_50_ values for HGSOC cell lines COV362 and OVCAR-4 and control cell line HIO80.

Cell line	HIO80		COV362		OVCAR-4	
Cpds	IC_50_ (μM)	95% Confidence Interval	IC_50_ (μM)	95% Confidence Interval	IC_50_ (μM)	95% Confidence Interval
**1**	41.88	29.64–55.74	96.59	67.48–139.1		
**2**	92.05	72.72–115.3	140.7	116.8–166.8		
**3**	34.72	20.85–52.73	90.22	70.12–124.2		
**5**	60.50	41.77–83.68	136.20	96.60–191.6		
**6**	187.3	162.0–215.4	151.50	131.7–171.9		
**7**	14.32	12.26–17.01	17.67	12.99–24.10	28.05	26.90–29.26
**8**	32.54	29.36–35.70	38.04	29.47–47.19	97.50	88.30–108.2
**9**	12.76	10.75–15.39	21.53	18.70–24.74	14.36	12.89–15.99
**10**	>200.00	NA	>200.00	NA		
Clioquinol			20.08	17.07–23.55		
Nitroxoline			6.60	5.72–7.53		
Cisplatin	6.96	5.50–8.74	13.55	11.27–16.67	4.16	2.61–6.13
Carboplatin	131.30	106.3–181.1	271.60	NA	73.02	60.40–85.15
RAPTA-C			>300	NA		

NA, not available as it cannot be accurately calculated.

**FIGURE 6:**
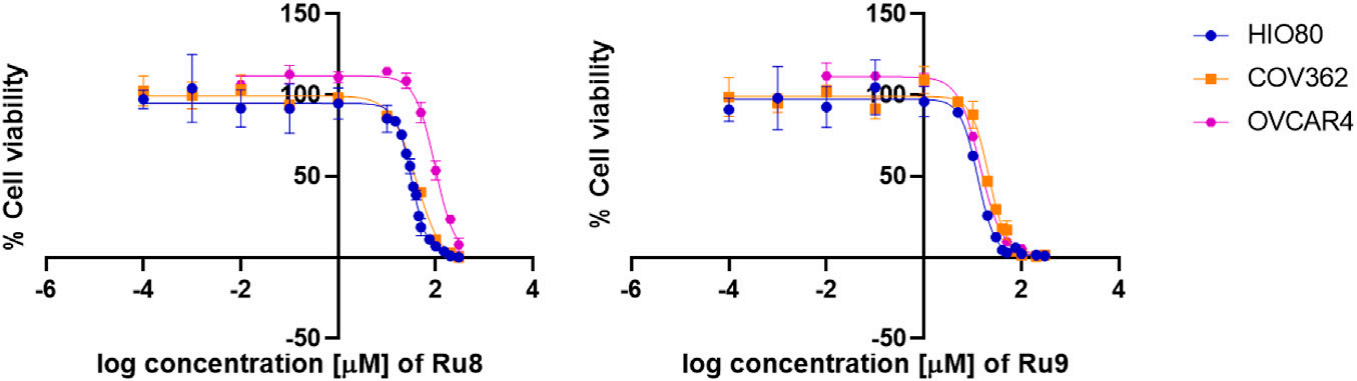
Anti-proliferative action of Ru complexes and platin-based drugs. Curves for determination of IC_50_ values are shown for Ru complexes **8** and **9**.

Similarly, cisplatin and carboplatin, currently used for the treatment of ovarian cancer, also showed a better effect on the control cell line HIO80 and the chemosensitive cell line OVCAR-4 with an approximately two to three-fold lower IC_50_ value for cisplatin (6.96 and 4.16 µM, respectively) and carboplatin (131.30 and 73.02 µM, respectively) as compared to the chemoresistant cell line COV362 (13.55 µM for cisplatin and 271.60 µM for carboplatin). We have to point out that all ruthenium complexes, except compound **10** shows a 1.8 to 4.6-fold better effects on proliferation of chemoresistant cell line COV362 compared to carboplatin and have a similar effect (with IC_50_ values in the same concentration range) compared to cisplatin (Figure 6; Supplementary Figure S3). In general, we can conclude that the presence of the hydroxyquinolinato and chlorido ligands increases the cytotoxic effect of the ruthenium species compared to their diketonato and pta counterparts with the exception of outlier compound **8**.

We also evaluated the effects of ligands clioquinol (ligand in complexes **7** and **8**) and nitroxoline (ligand in complexes **9** and **10**) and ruthenium complex RAPTA-C. Clioquinol showed similar toxicity compared to its Ru-chlorido complex **7** but slightly higher toxicity compared to its Ru-pta complex **8** with 1.9-fold lower IC_50_ value. Nitroxoline itself showed high cytotoxicity with 4-fold lower compared to its Ru-chlorido complex **9** while its Ru-pta complex **10** is inactive. This data shows that these ligands exert slightly higher antiproliferative action on chemoresistant cell line COV362 compared to their Ru complexes yet of the same order of magnitude. Nitroxoline was even more efficient than cisplatin. RAPTA-C expectedly showed almost no effect with IC_50_ value > 300 microM as it is known for its antimetastatic properties (Scolaro et al., 2005; Babak et al., 2015). We have to stress that all nine Ru complexes investigated in this study showed higher toxicity to the cancer cell line tested than RAPTA-C including all complexes bearing O,O-diketonate ligands.

**FIGURE 7:**
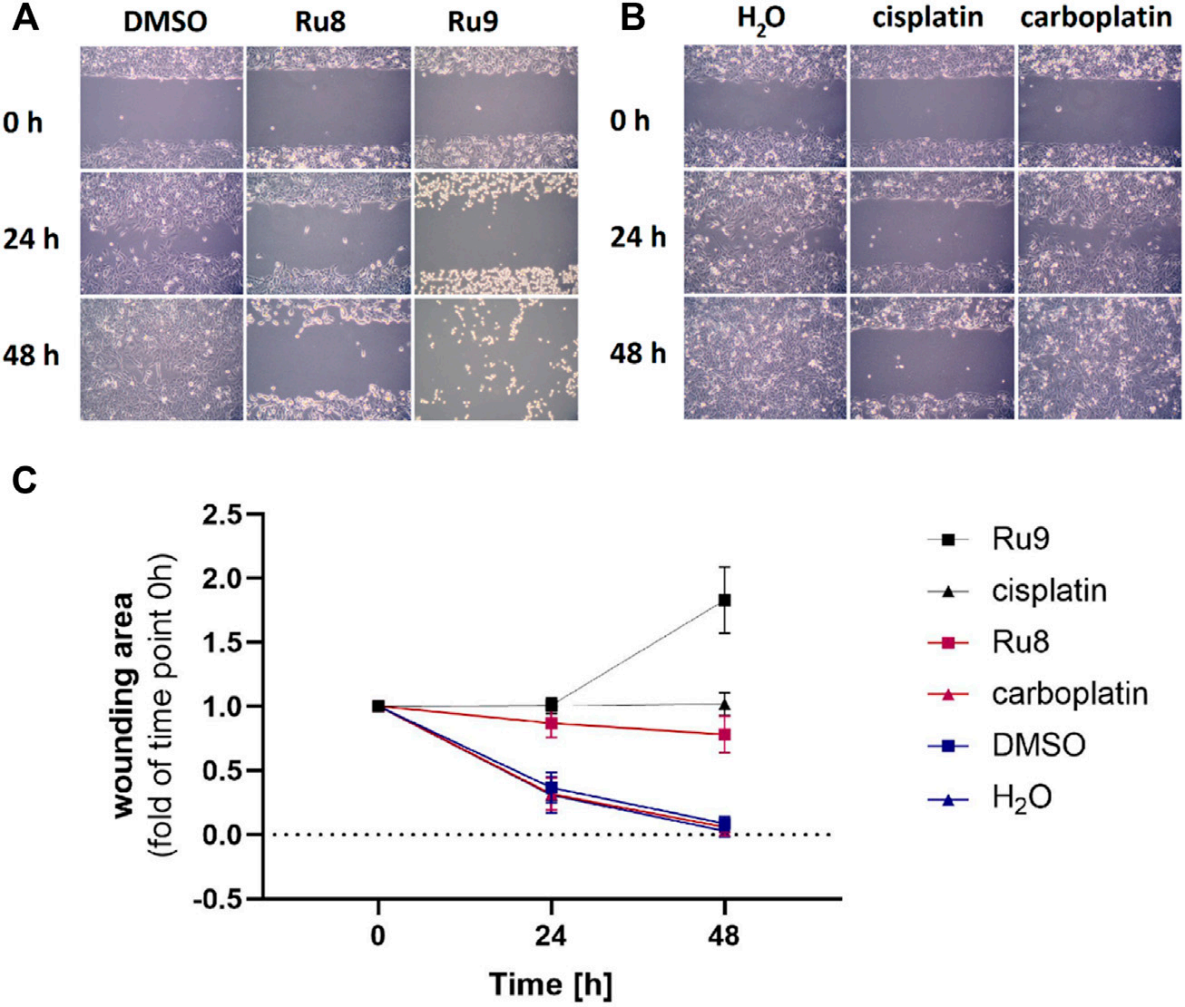
Ru complexes **8** and **9** affect migration of COV362 cells. Migration of COV362 cells in the presence of Ru complexes **8** and **9** **(A)**, cisplatin and carboplatin **(B)** after 24 and 48 h **(C)** changes in wounding area over time.

For the most potent anti-proliferative agents **8** and **9** from both structural groups, we examined also their effects on cell migration (Figure 7). Compounds **8** and **9** were tested in comparison to cisplatin, carboplatin, DMSO, and water as controls. Significant cell migration was observed in cells treated with carboplatin and in both controls where cells completely filled the wound after 48 h. Compound **8** inhibited cell migration similarly to cisplatin, while complex **9** had the greatest effect and prevented migration, however, it also showed a cytotoxic effect after 48 h incubation.

This data confirms that compounds **7** and **9** have good anti-cancer potential as they act almost as potently on chemoresistant high-grade serous ovarian cancer cell line COV-362 as cisplatin, while compounds **8** and **9** show better anti-proliferative and anti-migration effects as compared to carboplatin.

## 4 Conclusion

In summary, we report in this study the synthesis, physicochemical characterization, and crystal structures of three new organoruthenium complexes (**3**, **4**, **10**). Together with seven other complexes from our compound library, we investigated these compounds as potential agents against chemoresistant ovarian cancer. First, we investigated the inhibitory potency and molecular mode of action of these compounds against their potential targets, enzymes belonging to the family of aldo-keto reductases 1C, which are involved in the molecular mechanisms of chemoresistance in ovarian cancer. Our studies show that these compounds are potent inhibitors of AKR1C1-3 enzymes with an unusual inhibitory mechanism in which two inhibitor molecules bind to the enzyme in a first rapid and reversible step and a second slower and irreversible step. The binding strength of each step depends on the chemical structure of the monodentate ligands in the metalloinhibitors with the chlorido complexes generally acting as reversible inhibitors and the pta complexes as irreversible inhibitors. In addition, the choice of monodentate ligand generally results in specific selectivity in inhibitory potency toward different members of the AKR1C family. The reversible chlorido inhibitors are generally more efficient toward the AKR1C1 enzyme, whereas the irreversible pta inhibitors are about an order of magnitude less effective toward AKR1C3. The mode of action is based on the known chemistry of organoruthenium compounds under physiological conditions in combination with the analysis of structural data involving ruthenium compounds from the PDB database, the detailed kinetic study of the inhibitory mechanism and molecular dynamics simulations. Based on the collected data, we propose the rapid binding of an intact inhibitor molecule to the enzyme-NADH complex followed by the slower binding of the hydrolyzed inhibitor species. Second, we examined the effects of these Ru compounds on the proliferation and migration of a model cell line of high-grade serous ovarian cancer. Our study shows that compounds **7**, and **9** act as potently as cisplatin, and compounds **8** and **9** have better anti-proliferative and anti-migration effects as compared to carboplatin.

## Data Availability

The datasets presented in this study can be found in online repositories. The names of the repository/repositories and accession number(s) can be found below: https://www.ccdc.cam.ac.uk/; Deposition Numbers: 2063232-2063234.

## Supplementary material

The Supplementary Material for this study can be found online at: https://www.frontiersin.org/articles/10.3389/fphar.2022.920379/full#supplementary-material
