# Supplementary material for: Ruthenium complexes show potent inhibition of AKR1C1, AKR1C2, and AKR1C3 enzymes and anti-proliferative action against chemoresistant ovarian cancer cell line
Source: Front Pharmacol. 2022 Aug 11;13:920379. doi: 10.3389/fphar.2022.920379 (PMC9403717; doi:10.3389/fphar.2022.920379)

checkCIF/PLATON report

Structure factors have been supplied for datablock(s) cu420, cu652, moa537

THIS REPORT IS FOR GUIDANCE ONLY. IF USED AS PART OF A REVIEW PROCEDURE FOR PUBLICATION, IT SHOULD NOT REPLACE THE EXPERTISE OF AN EXPERIENCED CRYSTALLOGRAPHIC REFEREE.

No syntax errors found. CIF dictionary Interpreting this report

Datablock: moa537

```
Bond precision:  C-C = 0.0033 A          Wavelength=0.71073

Cell:          a=7.6996(3)       b=11.8327(5)     c=12.8523(5)
               alpha=83.018(3)   beta=77.340(3)   gamma=73.084(3)
Temperature:   150 K

                Calculated                 Reported
Volume          1090.94(8)                 1090.94(8)
Space group     P -1                       P -1
Hall group      -P 1                       -P 1
Moiety formula  C22 H25 Cl F3 N O2 Ru      C22 H25 Cl F3 N O2 Ru
Sum formula     C22 H25 Cl F3 N O2 Ru      C22 H25 Cl F3 N O2 Ru
Mr              528.95                     528.95
Dx,g cm-3       1.610                      1.610
Z               2                          2
Mu (mm-1)       0.885                      0.885
F000            536.0                      536.0
F000'           534.02
h,k,lmax        10,16,18                   10,16,16
Nref            6502                       5622
Tmin,Tmax       0.915,0.957                0.746,1.000
Tmin'           0.915

Correction method= # Reported T Limits: Tmin=0.746 Tmax=1.000
AbsCorr = MULTI-SCAN

Data completeness= 0.865          Theta(max)= 30.262

R(reflections)= 0.0330( 4701)     wR2(reflections)= 0.0599( 5622)

S = 0.997                  Npar= 276
```

The following ALERTS were generated. Each ALERT has the format
test-name_ALERT_alert-type_alert-level.
Click on the hyperlinks for more details of the test.

Alert level C

```
PLAT213_ALERT_2_C Atom F1A            has ADP max/min Ratio .....        3.2 prolat
PLAT910_ALERT_3_C Missing # of FCF Reflection(s) Below Theta(Min).         5 Note
```

Alert level G

```
PLAT154_ALERT_1_G The s.u.'s on the Cell Angles are Equal ..(Note)      0.003 Degree
PLAT912_ALERT_4_G Missing # of FCF Reflections Above STh/L=  0.600       795 Note
PLAT941_ALERT_3_G Average HKL Measurement Multiplicity ...........       1.7 Low
PLAT952_ALERT_5_G Calculated (ThMax) and CIF-Reported Lmax Differ          2 Units
PLAT958_ALERT_1_G Calculated (ThMax) and Actual (FCF) Lmax Differ          2 Units
PLAT978_ALERT_2_G Number C-C Bonds with Positive Residual Density.         7 Info
```

0 **ALERT level A** = Most likely a serious problem - resolve or explain
0 **ALERT level B** = A potentially serious problem, consider carefully
2 **ALERT level C** = Check. Ensure it is not caused by an omission or oversight
6 **ALERT level G** = General information/check it is not something unexpected

2 ALERT type 1 CIF construction/syntax error, inconsistent or missing data
2 ALERT type 2 Indicator that the structure model may be wrong or deficient
2 ALERT type 3 Indicator that the structure quality may be low
1 ALERT type 4 Improvement, methodology, query or suggestion
1 ALERT type 5 Informative message, check

Datablock: cu420

Bond precision: C-C = 0.0109 A Wavelength=1.54184

Cell:	a=11.9977(4)	b=16.2064(6)	c=16.7303(6)
	alpha=90	beta=100.767(3)	gamma=90
Temperature:	150 K		

	Calculated	Reported
Volume	3195.8(2)	3195.8(2)
Space group	P 21/n	P 1 21/n 1
Hall group	-P 2yn	-P 2yn
Moiety formula	C28 H37 F3 N4 O2 P Ru, F6 P	C28 H37 F3 N4 O2 P Ru, F6 P
Sum formula	C28 H37 F9 N4 O2 P2 Ru	C28 H37 F9 N4 O2 P2 Ru
Mr	795.63	795.62
Dx,g cm-3	1.654	1.654
Z	4	4
Mu (mm-1)	5.705	5.705
F000	1616.0	1616.0
F000'	1624.01	
h,k,lmax	14,19,20	14,19,20
Nref	6078	6060
Tmin,Tmax	0.789,0.843	0.286,1.000
Tmin'	0.716	

```
Correction method= # Reported T Limits: Tmin=0.286 Tmax=1.000
AbsCorr = MULTI-SCAN

Data completeness= 0.997          Theta(max)= 70.073

R(reflections)= 0.0640( 4779)     wR2(reflections)= 0.1860( 6060)

S = 1.041                  Npar= 418
```

The following ALERTS were generated. Each ALERT has the format
test-name_ALERT_alert-type_alert-level.
Click on the hyperlinks for more details of the test.

Alert level B

```
PLAT213_ALERT_2_B Atom C23            has ADP max/min Ratio .....        4.8 prolat
PLAT220_ALERT_2_B NonSolvent   Resd 1  C   Ueq(max)/Ueq(min) Range        8.4 Ratio
PLAT242_ALERT_2_B Low    'MainMol' Ueq as Compared to Neighbors of        C21 Check
```

Alert level C

```
PLAT213_ALERT_2_C Atom C18            has ADP max/min Ratio .....        3.8 prolat
PLAT222_ALERT_3_C NonSolvent Resd 1  H   Uiso(max)/Uiso(min) Range        8.9 Ratio
PLAT230_ALERT_2_C Hirshfeld Test Diff for    C21      --C22      .        5.5 s.u.
PLAT234_ALERT_4_C Large Hirshfeld Difference C18      --C19      .       0.19 Ang.
PLAT241_ALERT_2_C High   'MainMol' Ueq as Compared to Neighbors of        C18 Check
PLAT242_ALERT_2_C Low    'MainMol' Ueq as Compared to Neighbors of        Ru1 Check
PLAT250_ALERT_2_C Large U3/U1 Ratio for Average U(i,j) Tensor ....        2.3 Note
PLAT260_ALERT_2_C Large Average Ueq of Residue Including       P34      0.109 Check
PLAT342_ALERT_3_C Low Bond Precision on  C-C Bonds ..............    0.0109 Ang.
PLAT360_ALERT_2_C Short  C(sp3)-C(sp3) Bond  C21      - C22      .       1.40 Ang.
PLAT977_ALERT_2_C Check Negative Difference Density on H23A             -0.37 eA-3
```

Alert level G

```
PLAT002_ALERT_2_G Number of Distance or Angle Restraints on AtSite          3 Note
PLAT072_ALERT_2_G SHELXL First  Parameter in WGHT  Unusually Large       0.11 Report
PLAT083_ALERT_2_G SHELXL Second Parameter in WGHT  Unusually Large       6.79 Why ?
PLAT172_ALERT_4_G The CIF-Embedded .res File Contains DFIX Records          2 Report
PLAT242_ALERT_2_G Low    'MainMol' Ueq as Compared to Neighbors of         C1 Check
PLAT244_ALERT_4_G Low    'Solvent' Ueq as Compared to Neighbors of        P34 Check
PLAT380_ALERT_4_G Incorrectly? Oriented X(sp2)-Methyl Moiety .....        C12 Check
PLAT720_ALERT_4_G Number of Unusual/Non-Standard Labels ..........          3 Note
PLAT790_ALERT_4_G Centre of Gravity not Within Unit Cell: Resd.  #          2 Note
              F6 P
PLAT860_ALERT_3_G Number of Least-Squares Restraints .............          2 Note
PLAT912_ALERT_4_G Missing # of FCF Reflections Above STh/L=  0.600         19 Note
PLAT941_ALERT_3_G Average HKL Measurement Multiplicity ...........        2.1 Low
PLAT978_ALERT_2_G Number C-C Bonds with Positive Residual Density.          1 Info
```

---

0 **ALERT level A** = Most likely a serious problem - resolve or explain
3 **ALERT level B** = A potentially serious problem, consider carefully
11 **ALERT level C** = Check. Ensure it is not caused by an omission or oversight
13 **ALERT level G** = General information/check it is not something unexpected

0 ALERT type 1 CIF construction/syntax error, inconsistent or missing data

```
 16 ALERT type 2 Indicator that the structure model may be wrong or deficient
  4 ALERT type 3 Indicator that the structure quality may be low
  7 ALERT type 4 Improvement, methodology, query or suggestion
  0 ALERT type 5 Informative message, check
```

Datablock: cu652

```
Bond precision:  C-C = 0.0098 A         Wavelength=1.54184

Cell:            a=10.4749(4)       b=11.7911(4)      c=15.3720(8)
                 alpha=80.006(4)    beta=70.269(4)    gamma=64.035(4)
Temperature:     150 K
```

	Calculated	Reported
Volume	1606.00(13)	1606.00(13)
Space group	P -1	P -1
Hall group	-P 1	-P 1
Moiety formula	C25 H31 N5 O3 P Ru, F5 P, C4 H9 O	C25 H31 N5 O3 P Ru, C4 H9 O, F5 P
Sum formula	C29 H40 F5 N5 O4 P2 Ru	C29 H40 F5 N5 O4 P2 Ru
Mr	780.67	780.67
Dx,g cm-3	1.614	1.614
Z	2	2
Mu (mm-1)	5.537	5.537
F000	800.0	800.0
F000’	803.71	
h,k,lmax	12,14,18	12,14,18
Nref	6106	5290
Tmin,Tmax	0.304,0.330	0.368,1.000
Tmin’	0.095	

```
Correction method= # Reported T Limits: Tmin=0.368 Tmax=1.000
AbsCorr = MULTI-SCAN

Data completeness= 0.866          Theta(max)= 70.049

R(reflections)= 0.0570( 4971)     wR2(reflections)= 0.1599( 5290)

S = 1.083                  Npar= 462
```

The following ALERTS were generated. Each ALERT has the format
test-name_ALERT_alert-type_alert-level.
Click on the hyperlinks for more details of the test.

Alert level A

PLAT029_ALERT_3_A _diffrn_measured_fraction_theta_full value Low .   0.871 Why?

Alert level B

```
PLAT911_ALERT_3_B Missing FCF Refl Between Thmin & STh/L=    0.600        751 Report
```

Alert level C

```
CRYSC01_ALERT_1_C The word below has not been recognised as a standard
            identifier.
            dull
PLAT222_ALERT_3_C NonSolvent Resd 1  H   Uiso(max)/Uiso(min) Range        4.7 Ratio
PLAT234_ALERT_4_C Large Hirshfeld Difference P1       --F6       .       0.22 Ang.
PLAT234_ALERT_4_C Large Hirshfeld Difference P1       --F7       .       0.17 Ang.
PLAT234_ALERT_4_C Large Hirshfeld Difference P1       --F2A      .       0.22 Ang.
PLAT243_ALERT_4_C High   'Solvent' Ueq as Compared to Neighbors of        C41 Check
PLAT243_ALERT_4_C High   'Solvent' Ueq as Compared to Neighbors of        C43 Check
PLAT250_ALERT_2_C Large U3/U1 Ratio for Average U(i,j) Tensor ....        2.2 Note
PLAT260_ALERT_2_C Large Average Ueq of Residue Including        P1      0.125 Check
PLAT342_ALERT_3_C Low Bond Precision on  C-C Bonds ...............    0.00976 Ang.
PLAT362_ALERT_2_C Short  C(sp3)-C(sp2) Bond  C40      - C41      .       1.41 Ang.
PLAT918_ALERT_3_C Reflection(s) with I(obs) much Smaller I(calc) .          1 Check
PLAT972_ALERT_2_C Check Calcd Resid. Dens.  0.57A   From P1             -1.58 eA-3
PLAT972_ALERT_2_C Check Calcd Resid. Dens.  0.58A   From P1             -1.54 eA-3
```

Alert level G

```
PLAT042_ALERT_1_G Calc. and Reported MoietyFormula Strings  Differ     Please Check
PLAT072_ALERT_2_G SHELXL First  Parameter in WGHT  Unusually Large       0.11 Report
PLAT154_ALERT_1_G The s.u.'s on the Cell Angles are Equal ..(Note)      0.004 Degree
PLAT164_ALERT_4_G Nr. of Refined C-H H-Atoms in Heavy-Atom Struct.          1 Note
PLAT231_ALERT_4_G Hirshfeld Test (Solvent)   P1       --F1       .        6.8 s.u.
PLAT231_ALERT_4_G Hirshfeld Test (Solvent)   P1       --F2       .        6.0 s.u.
PLAT244_ALERT_4_G Low    'Solvent' Ueq as Compared to Neighbors of         P1 Check
PLAT302_ALERT_4_G Anion/Solvent/Minor-Residue Disorder (Resd  2  )        50% Note
PLAT302_ALERT_4_G Anion/Solvent/Minor-Residue Disorder (Resd  3  )        20% Note
PLAT412_ALERT_2_G Short Intra XH3 .. XHn     H44A     ..H43D     .       2.11 Ang.
                                                      x,y,z  =      1_555 Check
PLAT412_ALERT_2_G Short Intra XH3 .. XHn     H44B     ..H43C     .       2.09 Ang.
                                                      x,y,z  =      1_555 Check
PLAT432_ALERT_2_G Short Inter X...Y Contact  F7       ..C13              2.96 Ang.
                                                      x,y,z  =      1_555 Check
PLAT912_ALERT_4_G Missing # of FCF Reflections Above STh/L=  0.600         65 Note
PLAT913_ALERT_3_G Missing # of Very Strong Reflections in FCF ....          1 Note
PLAT941_ALERT_3_G Average HKL Measurement Multiplicity ...........        2.0 Low
PLAT978_ALERT_2_G Number C-C Bonds with Positive Residual Density.          0 Info
```

```
  1 ALERT level A = Most likely a serious problem - resolve or explain
  1 ALERT level B = A potentially serious problem, consider carefully
 14 ALERT level C = Check. Ensure it is not caused by an omission or oversight
 16 ALERT level G = General information/check it is not something unexpected

  3 ALERT type 1 CIF construction/syntax error, inconsistent or missing data
 10 ALERT type 2 Indicator that the structure model may be wrong or deficient
  7 ALERT type 3 Indicator that the structure quality may be low
 12 ALERT type 4 Improvement, methodology, query or suggestion
  0 ALERT type 5 Informative message, check
```

It is advisable to attempt to resolve as many as possible of the alerts in all categories. Often the minor alerts point to easily fixed oversights, errors and omissions in your CIF or refinement strategy, so attention to these fine details can be worthwhile. In order to resolve some of the more serious problems it may be necessary to carry out additional measurements or structure refinements. However, the purpose of your study may justify the reported deviations and the more serious of these should normally be commented upon in the discussion or experimental section of a paper or in the "special_details" fields of the CIF. checkCIF was carefully designed to identify outliers and unusual parameters, but every test has its limitations and alerts that are not important in a particular case may appear. Conversely, the absence of alerts does not guarantee there are no aspects of the results needing attention. It is up to the individual to critically assess their own results and, if necessary, seek expert advice.

Publication of your CIF in IUCr journals

A basic structural check has been run on your CIF. These basic checks will be run on all CIFs submitted for publication in IUCr journals (*Acta Crystallographica*, *Journal of Applied Crystallography*, *Journal of Synchrotron Radiation*); however, if you intend to submit to *Acta Crystallographica Section C* or *E* or *IUCrData*, you should make sure that full publication checks are run on the final version of your CIF prior to submission.

Publication of your CIF in other journals

Please refer to the *Notes for Authors* of the relevant journal for any special instructions relating to CIF submission.

PLATON version of 05/12/2020; check.def file version of 05/12/2020

Datablock moa537 - ellipsoid plot

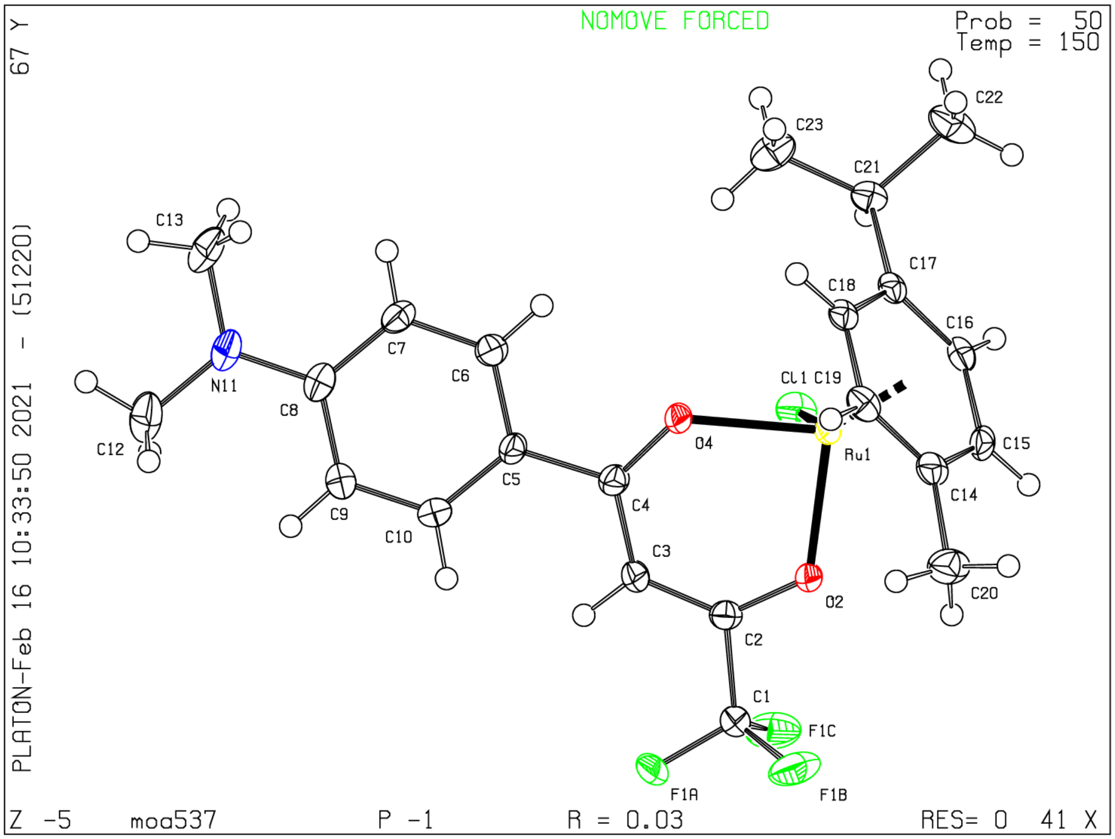

Datablock cu420 - ellipsoid plot

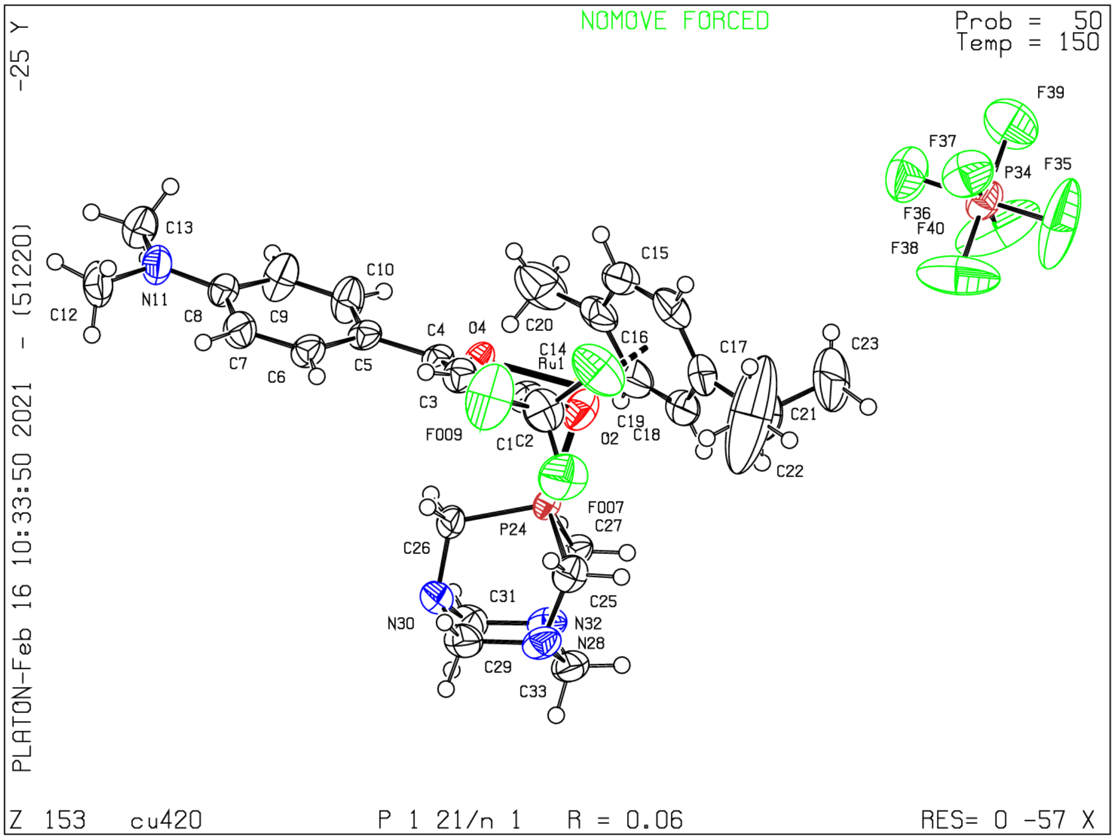

Datablock cu652 - ellipsoid plot

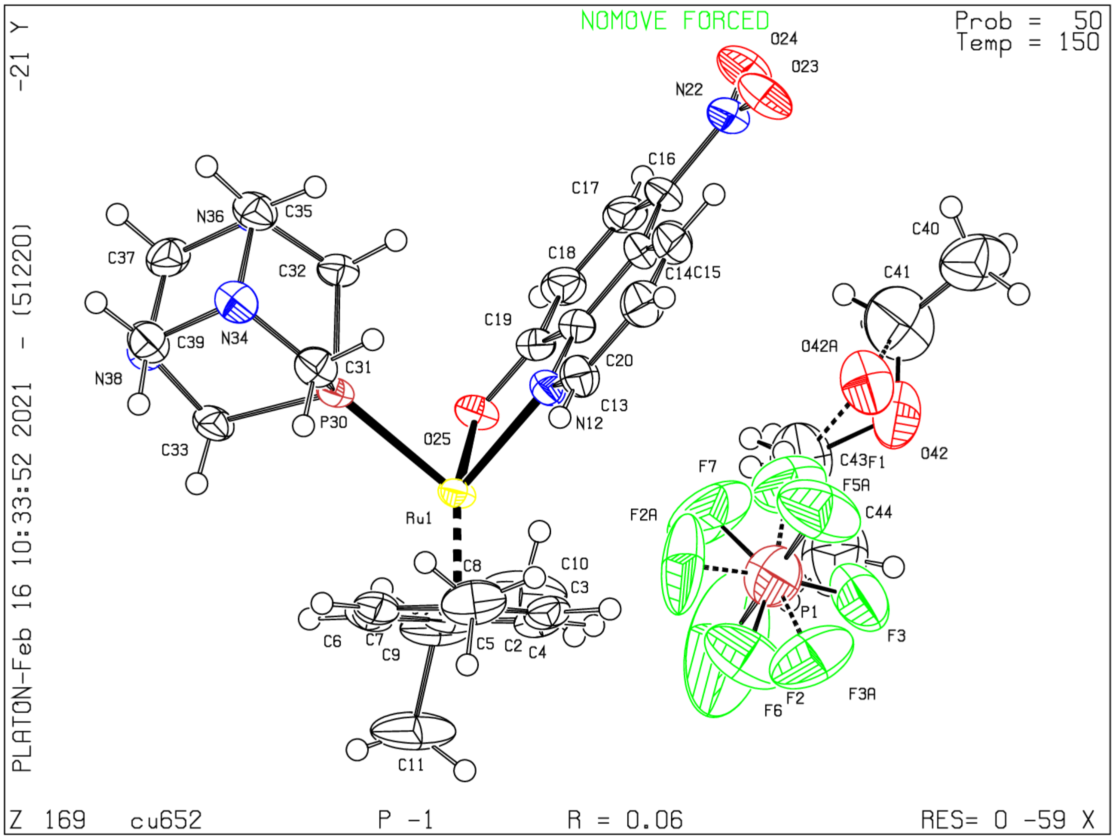

Supplement: Supplementary file 1 [file DataSheet2.pdf]
