# Supplementary material for: Ruthenium complexes show potent inhibition of AKR1C1, AKR1C2, and AKR1C3 enzymes and anti-proliferative action against chemoresistant ovarian cancer cell line
Source: Front Pharmacol. 2022 Aug 11;13:920379. doi: 10.3389/fphar.2022.920379 (PMC9403717; doi:10.3389/fphar.2022.920379)

Ruthenium complexes show potent inhibition of AKR1C1, AKR1C2 and AKR1C3 enzymes and anti-proliferative action against chemoresistant ovarian cancer cell line

# Supplementary information file

Jakob Kljun,^1^ Renata Pavlič,^2^ Eva Hafner^2^, Tanja Lipec,^1^ Sara Moreno Da Silva,^1,3^ Primož Tič,^1^ Iztok Turel^1,^* Tomaž Büdefeld,^2^ Jure Stojan^2^ and Tea Lanišnik Rižner^2,^*

^1^ University of Ljubljana, Faculty of Chemistry and Chemical Technology, Department of Chemistry and Biochemistry, Večna pot 113, SI-1000 Ljubljana, Slovenia

^2^ University of Ljubljana, Faculty of Medicine, Institute of Biochemistry, Vrazov trg 2, SI-1000 Ljubljana, Slovenia

^3^ Universidad Complutense de Madrid, Faculty of Chemical Sciences, Av. Complutense, s/n, 28040 Madrid, Spain

Contents of SI:

**Table S1**: Crystallographic data for compounds **3**, **4**, and **10**.

**Figure S1:** Photographs of analysed crystals of compounds **3**, **4**, **10**.

**Figures S2(.1-.10):** Progress curves showing inhibition of AKR1C enzymes by Ru complexes studied.

**Figure S3:** Anti-proliferative action of Ru complexes and platinum based drugs. Curves for determination of IC_50_ values are shown for Ru complex **7**, cisplatin and carboplatin.

**Table S1**: Crystallographic data for compounds **3**, **4**, and **10**.

| Compound | **3** | **4** | **10·Et_2_O** |
| --- | --- | --- | --- |
| Empirical formula | C_22_H_25_ClF_3_NO_2_Ru | C_28_H_37_F_9_N_4_O_2_P_2_Ru | C_29_H_40_F_5_N_5_O_4_P_2_Ru |
| Formula weight | 528.95 | 795.62 | 780.67 |
| Temperature/K | 150 | 150 | 150 |
| Crystal system | triclinic | monoclinic | triclinic |
| Space group | P-1 | P2_1_/n | P-1 |
| a/Å | 7.6996(3) | 11.9977(4) | 10.4749(4) |
| b/Å | 11.8327(5) | 16.2064(6) | 11.7911(4) |
| c/Å | 12.8523(5) | 16.7303(6) | 15.3720(8) |
| α/° | 83.018(3) | 90 | 80.006(4) |
| β/° | 77.340(3) | 100.767(3) | 70.269(4) |
| γ/° | 73.084(3) | 90 | 64.035(4) |
| Volume/Å^3^ | 1090.94(8) | 3195.8(2) | 1606.00(13) |
| Z | 2 | 4 | 2 |
| ρ_calc_g/cm^3^ | 1.610 | 1.654 | 1.614 |
| μ/mm^‑1^ | 0.885 | 5.705 | 5.537 |
| F(000) | 536.0 | 1616.0 | 800.0 |
| Crystal size/mm^3^ | 0.1 × 0.1 × 0.05 | 0.05 × 0.05 × 0.03 | 0.4 × 0.2 × 0.2 |
| Radiation | MoKα (λ = 0.71073) | CuKα (λ = 1.54184) | CuKα (λ = 1.54184) |
| 2Θ range for data collection/° | 5.804 – 60.524 | 7.66 – 140.146 | 6.112 to 140.098 |
| Index ranges | -10 ≤ h ≤ 10,  -10 ≤ k ≤ 16,  -15 ≤ l ≤ 16 | -12 ≤ h ≤ 14,  -14 ≤ k ≤ 19,  -20 ≤ l ≤ 18 | -10 ≤ h ≤ 12,  -9 ≤ k ≤ 14,  -18 ≤ l ≤ 18 |
| Reflections collected | 9791 | 12554 | 10737 |
| Independent reflections | 5622 [R_int_ = 0.0281, R_sigma_ = 0.0604] | 6060 [R_int_ = 0.0428, R_sigma_ = 0.0479] | 5290 [R_int_ = 0.0337, R_sigma_ = 0.0342] |
| Data/restraints/parameters | 5622/0/276 | 6060/2/418 | 5290/0/462 |
| Goodness-of-fit on F^2^ | 0.997 | 1.041 | 1.083 |
| Final R indexes [I>=2σ (I)] | R_1_ = 0.0330,  wR_2_ = 0.0565 | R_1_ = 0.0640,  wR_2_ = 0.1684 | R_1_ = 0.0570,  wR_2_ = 0.1556 |
| Final R indexes [all data] | R_1_ = 0.0453,  wR_2_ = 0.0599 | R_1_ = 0.0800,  wR_2_ = 0.1860 | R_1_ = 0.0596,  wR_2_ = 0.1599 |
| Largest diff. peak/hole / e Å^-3^ | 0.44/-0.48 | 1.18/-1.43 | 0.80/-1.58 |


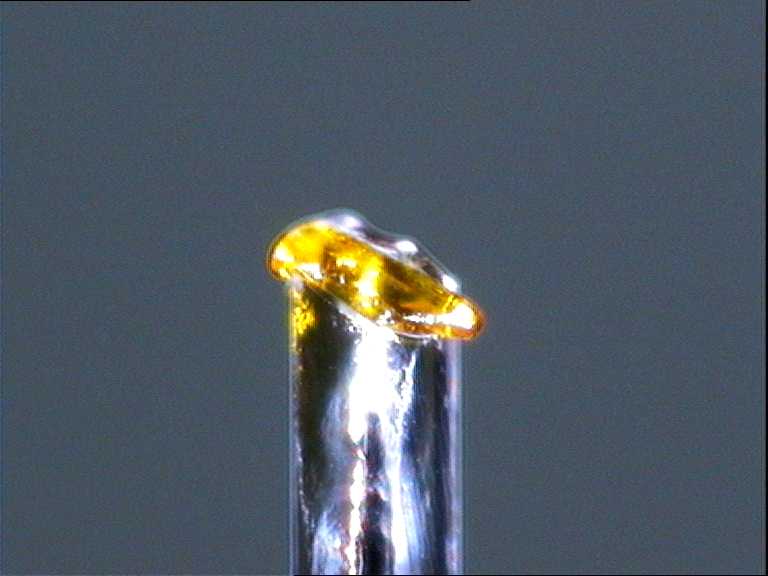

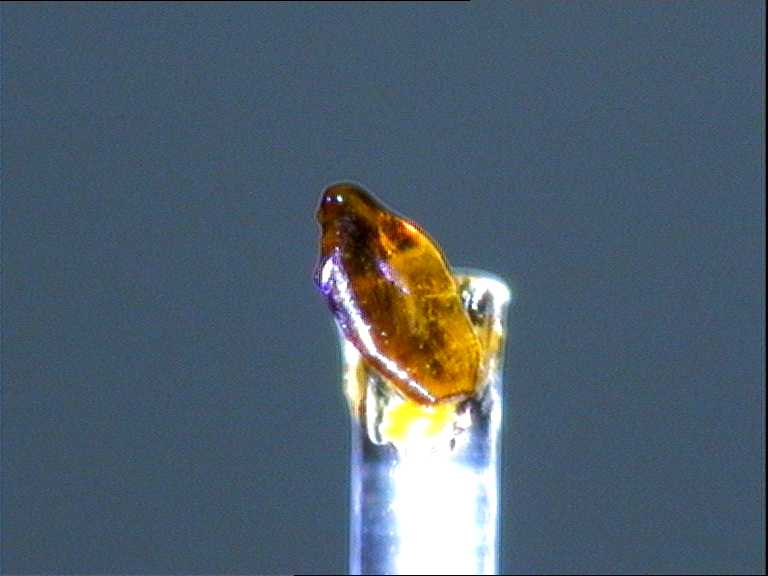

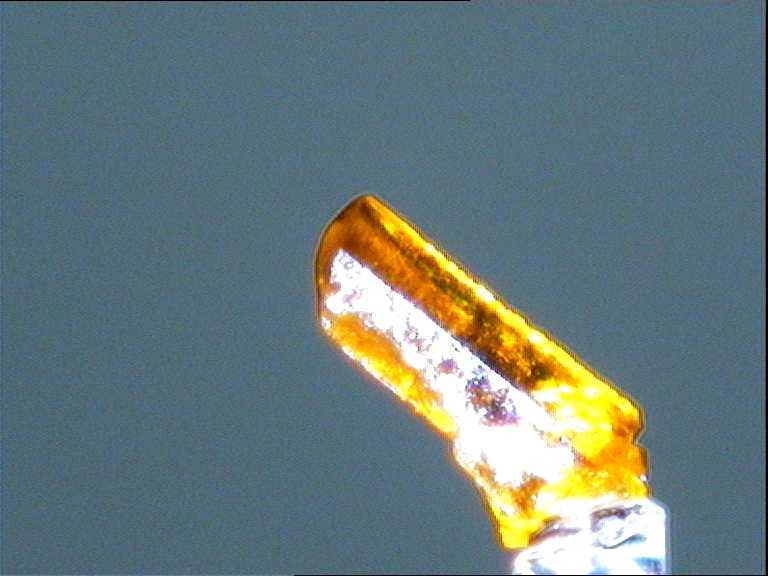


**Figure S1:** Photographs of analysed crystals of compounds **3**, **4**, **10**.

**Figures S2(.1-.10): Progress curves showing inhibition of AKR1C enzymes by Ru complexes studied.**

| Figure S2.1: Ru-complex **1** |  |  |
| --- | --- | --- |
|  |  |  |
|  |  |  |
|  |  |  |

| Figure S2.2: Ru-complex **2** |  |  |
| --- | --- | --- |
|  |  |  |
|  |  |  |
|  |  |  |

| Figure S2.3: Ru-complex **3** |  |  |
| --- | --- | --- |
|  |  |  |
|  |  |  |
|  |  |  |

| Figure S2.4: Ru-complex **4** |  |  |
| --- | --- | --- |
|  |  |  |
|  |  |  |
|  |  |  |
|  |  |  |

| Figure S2.5: Ru-complex **5** |  |  |
| --- | --- | --- |
|  |  |  |
|  |  |  |
|  |  |  |

| Figure S2.6: Ru-complex **6** |  |  |
| --- | --- | --- |
|  |  |  |
|  |  |  |
|  |  |  |

| Figure S2.7: Ru-complex **7** |  |  |
| --- | --- | --- |
|  |  |  |
|  |  |  |
|  |  |  |

| Figure S2.8: Ru-complex **8** |  |  |
| --- | --- | --- |
|  |  |  |
|  |  |  |
|  |  |  |
|  |  |  |

| Figure S2.9: Ru-complex **9** |  |  |
| --- | --- | --- |
|  |  |  |
|  |  |  |
|  |  |  |

| Figure S2.10: Ru-complex **10** | |  |
| --- | --- | --- |
|  | |  |
|  |  |  |
|  |  |  |

**Figure S3: Anti-proliferative action of Ru complexes and platinum based drugs.** Curves for determination of IC_50_ values are shown for Ru complex **7, cisplatin and carboplatin**.


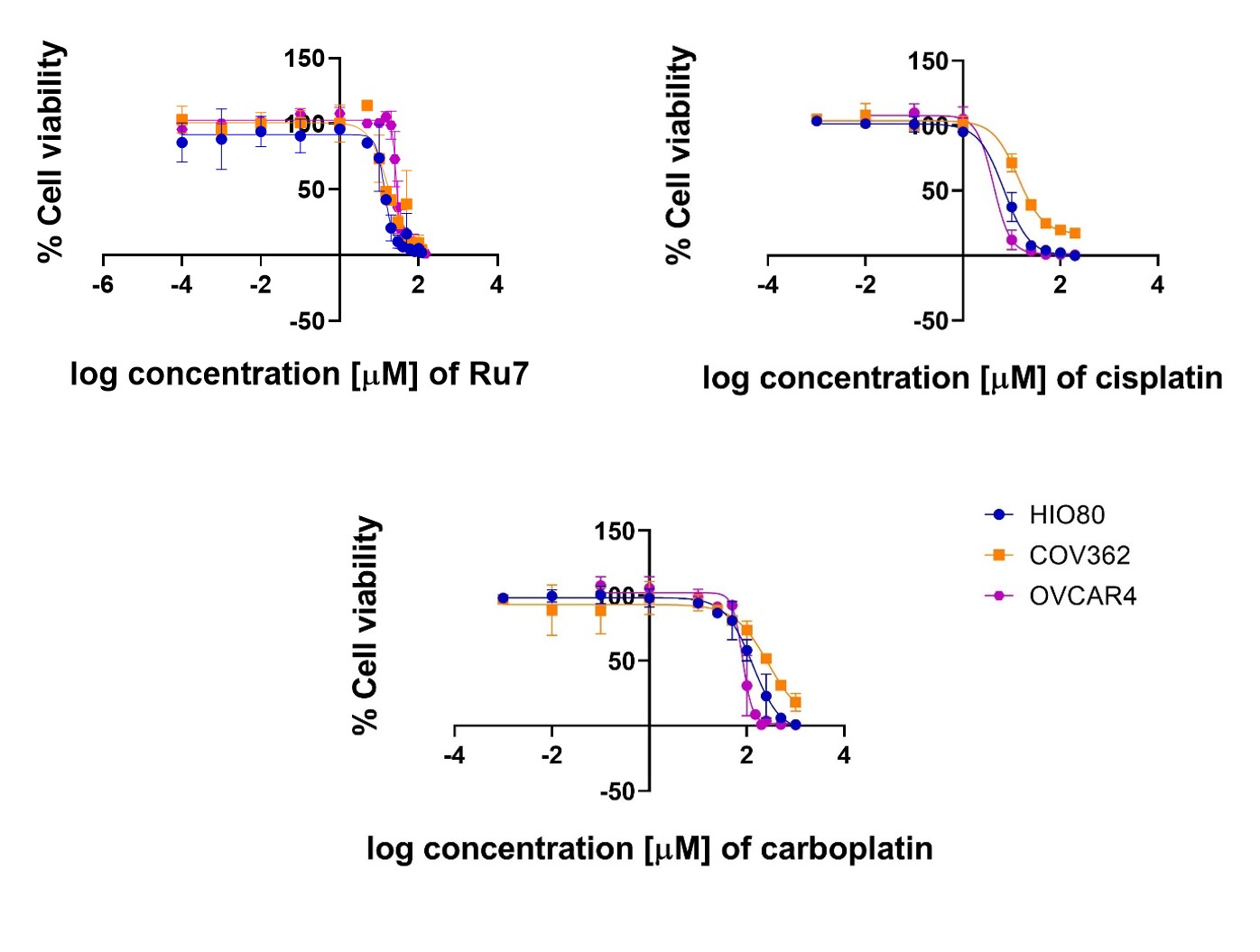

Supplement: Supplementary file 5 [file DataSheet1.docx]
